# Toward Sustainable Green and Intelligent Profile Control Gels: An ETI–CFI-Based Structure–Environment Evaluation Framework

**DOI:** 10.3390/gels11120952

**Published:** 2025-11-27

**Authors:** Qiang Chen, Hanmin Xiao, Zhihua Chen, Tong Wu, Hao Chen, Keqiang Wei

**Affiliations:** 1 University of Chinese Academy of Sciences, Beijing 100049, China; 2 Institute of Porous Flow and Fluid Mechanics, Chinese Academy of Sciences, Langfang 065007, China; 3 State Key Laboratory of Enhanced Oil Recovery, Research Institute of Petroleum Exploration and Development, PetroChina, Beijing 100083, China; 4 School of Energy and Mechanical Engineering, Jiangxi University of Science and Technology, Nanchang 330013, China

**Keywords:** profile control gels, green polymer gels, intelligent responsive gels, Environmental Toxicity Index (ETI), Carbon Footprint Intensity (CFI), enhanced oil recovery (EOR), low-carbon transition

## Abstract

In the context of the “dual-carbon” strategy and the escalating challenges posed by ultra-high water-cut reservoirs, the development of green and intelligent profile control gels (PCGs) has become essential for balancing enhanced oil recovery (EOR) efficiency with environmental sustainability. In this study, a green performance evaluation framework integrating the Environmental Toxicity Index (ETI) and Carbon Footprint Intensity (CFI) is established to quantitatively assess the environmental friendliness of polymer gel systems. Representative gel types—including conventional chromium(III)–polyacrylamide(Cr(III)–PAM), citric acid–chitosan, and pH-responsive nanogels—are evaluated to reveal their structure–environment interactions. Comparative analysis shows that the Cr(III)–PAM system exhibits strong plugging capability but imposes the highest environmental burden (ETI = 1.45; CFI = 9.1 kg CO_2_e/kg), whereas the citric acid–chitosan system significantly reduces both toxicity (ETI = 0.42) and carbon footprint (CFI = 2.1). Meanwhile, pH-responsive nanogels demonstrate superior reservoir stability and sustainability under harsh conditions. The proposed ETI–CFI evaluation framework not only enables quantitative benchmarking of green performance but also provides a unified criterion for molecular design, material screening, and engineering application of intelligent green gels. This framework offers practical guidance for the low-carbon transformation of oilfield chemical systems, aligning innovation with sustainability objectives and supporting the realization of dual-carbon goals.

## 1. Introduction

**Figure 1:**
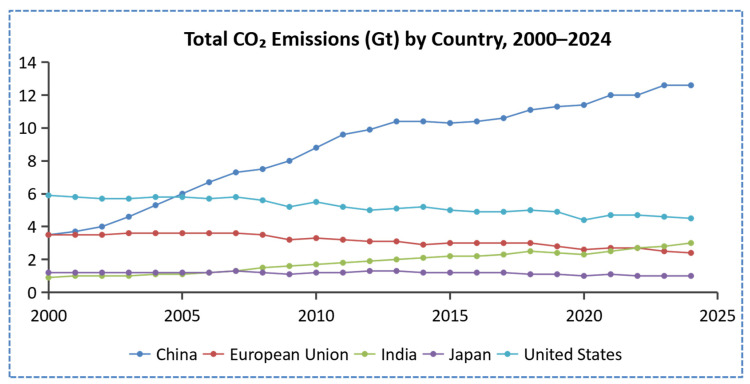
Global carbon dioxide (CO_2_) emissions [9].

Global energy-related CO_2_ emissions have continued to rise over the past two decades, reflecting the persistent reliance of industrial and energy systems on fossil fuels. As shown in Figure 1, CO_2_ emissions from major economies—including China, the United States, India, and the European Union—exhibit distinct trajectories: China’s rapid industrial expansion has driven the steepest growth since 2005, whereas emissions in the EU and Japan have gradually declined [1,2,3,4].

Complementing these trends, the IPCC AR6 WGIII Summary and the Global Carbon Budget 2023 indicate that cumulative anthropogenic CO_2_ emissions since the pre-industrial era exceed ~2500 Gt CO_2_, while the remaining carbon budget consistent with a 1.5 °C pathway has declined to <~300 Gt CO_2_ [5,6]. According to the International Energy Agency (IEA), global fossil-energy-related CO_2_ emissions reached 37.4 Gt in 2024, and more than 130 countries have incorporated carbon-reduction or carbon-neutrality targets into their national strategies [7,8]. This accelerating decarbonization momentum is reshaping the oil and gas sector toward low-carbon, environmentally adaptive technologies, such as polymer gels designed for carbon-efficient enhanced oil recovery (EOR). Nevertheless, despite rapid policy advances, the oil and gas industry remains among the most carbon-intensive sectors; process-level energy use and produced-water management are key determinants of overall carbon performance.

Under the dual-carbon framework, performance metrics in oilfield development are shifting from absolute hydrocarbon output to Carbon Footprint Intensity (CFI)—defined as life cycle CO_2_ emissions per unit of crude oil produced. In line with ISO 14040/14044 [10] and ISO 14067 [11], CFI is quantified within a life cycle assessment (LCA) framework that specifies the goal-and-scope definition [12,13], system boundaries, and life cycle inventory (LCI) compilation. Impact assessment can then be performed using standard LCIA method families—such as ReCiPe [14,15], USEtox for human/ecotoxicity [16,17,18,19,20], or TRACI for North American contexts [21,22], thereby ensuring methodological consistency and comparability. This shift in evaluation standards elevates energy use, chemical consumption, and produced-water management from secondary operational concerns to core elements of the carbon-reduction agenda. As a result, the operational inefficiencies of mature oilfields—particularly the excessive energy and water circulation associated with high water-cut production—have become key barriers to achieving carbon-intensity mitigation under the dual-carbon framework. Mature reservoirs operating at ultra-high water cut are major contributors to elevated CFI, as each additional barrel of injected or produced water increases injection pressure, lifting power, and treatment load while yielding little incremental oil. Accordingly, water management serves not merely as an operational efficiency measure but as a direct lever for carbon-intensity reduction in aging reservoirs.

As a result of such water-dominated operations, many mature fields experience severe heterogeneity due to prolonged waterflooding [23,24,25,26]. To sustain production under these conditions, operators are forced to increase injection rates and processing power, leading to a feedback cycle of higher energy demand, increased chemical consumption, and elevated CFI. Thus, the challenges of mature reservoirs—high water cut, poor sweep efficiency, and escalating energy intensity—are inherently intertwined with the dual-carbon goals. Reducing ineffective water cycling and restoring displacement conformance have, therefore, become necessary preconditions for reconciling stable production with low-carbon development, rather than optional efficiency upgrades.

Profile control and water-shutoff technologies have been widely deployed to mitigate reservoir heterogeneity and reduce ineffective water production. Among these, polymer gels—owing to their tunable three-dimensional networks and controllable plugging capacity—play a pivotal role in selectively sealing high-permeability channels and improving sweep efficiency [25,27]. Water-shutoff treatments are primarily used for local sealing of dominant water-producing zones [28,29,30,31,32], whereas profile control applications aim to enhance volumetric sweep efficiency and are recognized as a core pathway for improving oil recovery [33,34]. Critically, under dual-carbon constraints, effective profile control lowers the water–oil ratio (WOR), reduces lifting and injection energy demand, decreases produced-water treatment volume, and hence helps to reduce Carbon Footprint Intensity (CFI) while improving recovery efficiency. However, conventional chemical systems—such as Cr(III)–PAM and organic crosslinked gels—exhibit a trade-off between plugging performance and environmental compatibility, which limits their suitability for low-carbon development strategies [35,36].

In recent years, emerging environmentally friendly materials—including biopolymer gels, nanocomposite gels, and multi-responsive intelligent gels—have demonstrated promising adaptability and regulatory potential in complex reservoir conditions [37]. Nevertheless, large-scale deployment still faces three critical bottlenecks: (i) fragmented evaluation methodologies, (ii) limited mechanistic understanding under coupled geological–fluid–mechanical environments, and (iii) insufficient compatibility between low-carbon materials and existing injection–production systems. These limitations manifest as structural instability, inadequate thermal/saline resistance, and insufficient consideration of degradability and life cycle environmental impact.

**Figure 2:**
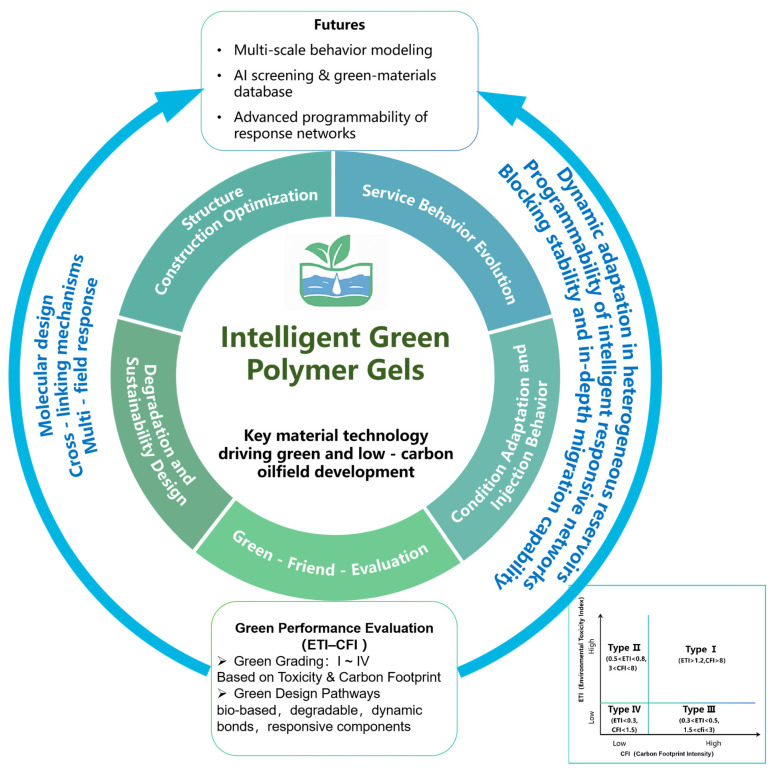
Framework of intelligent green polymer gels: construction dimensions and green evolution pathways.

Overcoming these challenges requires the development of intelligent green polymer gels within a systematic framework that integrates structure design, service behavior, and sustainability. To this end, this study introduces a green grading framework based on the Environmental Toxicity Index (ETI) and Carbon Footprint Intensity (CFI). The ETI is defined as a quantitative measure of the overall ecological hazard potential of a material, derived from weighted toxicity coefficients of its constituents toward aquatic and terrestrial organisms. The CFI represents the total greenhouse gas emissions, expressed as carbon dioxide equivalent (CO_2_e), associated with the life cycle production and application of the material per unit mass. The definitions, normalization rules, and scoring procedures for ETI and CFI are detailed in Section 2 (Equations (1)–(3)), including system boundaries and data sources. Figure 2 illustrates the conceptual framework, emphasizing the structural construction dimensions and green-evolution pathways of intelligent green gels.

Given these challenges, a systematic and quantitative approach is required to evaluate the environmental performance of polymer gel systems and to guide their green transformation. Building on this foundation, polymer gels are classified into four categories—conventional, low-toxicity, environmentally friendly, and intelligent green gels (Table 1). The structural optimization and environmental adaptation mechanisms of each type are systematically analyzed, with particular emphasis on the advances of intelligent responsive gels in reservoir heterogeneity identification, dynamic plugging regulation, and multi-field coupling performance. This work aims to provide theoretical support and engineering pathways for achieving the target of chemical-flooding carbon intensity ≤1.8 t CO_2_e m^−3^, as specified in the Specification for Green Mine Construction in Oilfield Development (SY/T 6788-2020) [38].

## 2. Results and Discussion

### 2.1. Development and Bottlenecks of Mainstream Gel Systems

With the continuous advancement of enhanced oil recovery (EOR) technologies, polymer gels have become a cornerstone in profile control and water-shutoff operations due to their tunable three-dimensional network structures and controllable plugging capability [55,56,57]. Despite extensive field applications, mainstream gel systems still face significant limitations under increasingly complex reservoir conditions. Their long-term performance is primarily constrained by three interrelated factors:

(i) Limited environmental tolerance: Most conventional gels cannot remain stable under complex reservoir conditions such as high temperature, high salinity, or variable pH environments, often leading to gel syneresis, premature degradation, or insufficient plugging radius [58,59,60].
(ii) High ecological risk: Systems based on Cr(III), Cr(Ⅵ), or phenol–formaldehyde organic crosslinkers exhibit inherent toxicity and are increasingly restricted by regulations such as the EU REACH directive [61] and China’s Measures for the Environmental Management of New Chemical Substances [62,63,64].
(iii) Weak dynamic responsiveness: Conventional gels are typically designed for static conditions, with limited adaptability to evolving seepage structures, resulting in rapid decline of plugging efficiency during long-term reservoir development [65,66,67].

To address these challenges, recent research has shifted toward reconstructing crosslinking mechanisms and optimizing gel-network architectures, promoting the transition from conventional gels to greener, more controllable, and intelligent systems. The following subsections review the evolution and major bottlenecks of these technologies from three perspectives: metal-ion crosslinked gels, organic crosslinked gels, and dynamic plugging failure mechanisms.

#### 2.1.1. Transition-Metal Gels: Structural Optimization and Ecological Risks

Metal-ion crosslinked gels form stable three-dimensional (3D) networks through coordination between multivalent cations and the carboxyl or hydroxyl groups of polymer chains [68]. Since the development and field deployment of Cr(III)–HPAM systems in the 1970s, these gels have been extensively used for deep-profile control and reservoir heterogeneity regulation, becoming one of the dominant plugging materials in tertiary oil recovery [69]. Nevertheless, they still face intrinsic drawbacks—rapid gelation kinetics, limited structural durability, and the ecological toxicity of heavy-metal species (notably Cr(Ⅵ))—which constrain their large-scale application and hinder their green transition.

Recent studies, therefore, aim to achieve a dual improvement in structural stability and environmental compatibility by optimizing both polymer backbones and crosslinking architectures. Under high-temperature and high-salinity (HT/HS) conditions, metal-ion gels typically undergo chain scission, ion shielding, and viscosity loss, motivating the development of multi-level reinforcement strategies. Three representative approaches have emerged: molecular modification, interfacial crosslink regulation, and nanocomposite reinforcement [45,70,71,72,73].

(i) **Backbone modification for thermo–salt resistance.** Incorporating hydrophobic monomers (e.g., styrene and alkyl acrylates) induces intra- and intermolecular associations, whereas grafting polar monomers (e.g., AMPS, NVP, and guanidinium) enhances chain rigidity and hydration stability [74,75,76,77,78,79]. For instance, Gumerov et al. [80] confirmed via DPD simulations that amphiphilic PVCL/TBCHA microgels with higher hydrophobic content exhibit stronger nanoscale interfacial bonding and improved thermo-responsive resilience. Similarly, Sarsenbekuly et al. [81] developed a hydrophobically modified polyacrylamide (RH-4) that maintained viscosity under 80,000 mg L^−1^ salinity, and Yang et al. [82] reported amphiphilic polymers maintaining > 90% volumetric stability and minimal shrinkage at 120 °C and salinity of 1.5 × 10^5^ mg/L. These results verify that molecular structure engineering is key to achieving thermo-salt-resistant backbones.
(ii) **Interfacial crosslink regulation for mechanical reinforcement.** Beyond backbone stabilization, fine-tuning of crosslink density and topology effectively improves mechanical integrity. Host–guest inclusion and branched architectures enhance network compactness and self-recovery. For example, β-cyclodextrin inclusion increased crosslink density and gel strength even at low dosage in [83]; grafting acrylamide onto CMC backbones yielded rigid frameworks with >30% branching degree in [84]; and Biswas et al. [78] achieved multilayer amphiphilic gels through sequential FRP–ATRP polymerization, where interlayer covalent coupling endowed high strength and elasticity. Collectively, these studies highlight that hierarchical polymer design, rather than single-component modification, dictates 3D network deformability under HT/HS stress.
(iii) **Nanocomposite reinforcement for structural durability.** To further compensate for backbone fragility, rigid–flexible hybridization using nanoparticles has gained attention. Inorganic fillers such as SiO_2_, montmorillonite, and cellulose nanocrystals impart rigidity and thermal resistance, while flexible polymers (e.g., PEG and polyglutamic acid) provide elasticity and interfacial adhesion [85,86]. Recent studies have confirmed the universality of this strategy across multiple systems. Sarvesh et al. [87] incorporated Laponite^®^ nanoclay into ABA-type PLA–PEO–PLA hydrogels, achieving nearly an order-of-magnitude increase in storage modulus. Das et al. [88] introduced graphene nanosheets into PAM matrices to enhance tensile strength, and Yang et al. [89] fabricated core–shell SiO_2_–PAM nanocomposites via in situ polymerization, markedly improving fracture strength. Hyperbranched silica nanoparticles (HBSPs) further reduced network density while preserving high deformability [90]. These results confirm that nano–macro-hierarchical coupling—rather than the mere addition of fillers—governs the enhancement of strength, elasticity, and thermal stability in metal-ion gels. Nevertheless, achieving uniform dispersion and interfacial compatibility at high nanoparticle loadings remains challenging, as agglomeration often deteriorates performance and limits large-scale application.

In summary, molecular modification, functional group grafting, and nanocomposite reinforcement have substantially improved the service stability and HT/HS tolerance of transition-metal gels (representative formulations are listed in Table 2). Despite these advances, current systems still rely predominantly on single-mechanism reinforcement, without establishing a unified structure–performance–environment correlation model. Moreover, the poor degradability of hydrophobic monomers and the agglomeration of nanoparticles at high concentrations continue to impair interfacial compatibility and dispersion stability, posing key challenges for large-scale and sustainable applications.

Building upon the advances in structural reinforcement, recent research has shifted its focus from purely performance-oriented design toward environmentally responsible gel systems. Despite significant improvements in mechanical integrity and HT/HS tolerance, conventional transition-metal crosslinkers—particularly chromium-based species [100,101]—pose persistent ecological and toxicological concerns due to their high mobility and oxidation potential [102,103]. Consequently, the green substitution of crosslinking systems has emerged as a critical pathway to achieve dual objectives: maintaining network strength while mitigating environmental hazards. This section, therefore, discusses the current strategies for green substitution and ecological risk reduction, with emphasis on (i) low-toxicity ligand chelation and (ii) multivalent-metal replacement.

**Figure 3:**
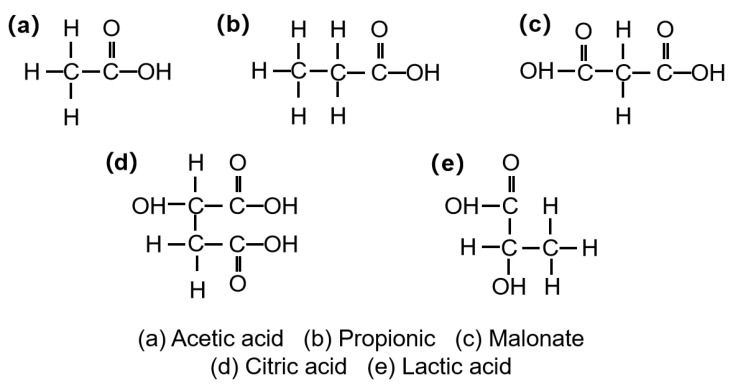
Molecular structures of typical organic acid ligands used for metal-ion coordination in polymer gel systems (structures drawn by the authors based on [45]).

(1) **Low-toxicity ligand chelation.** Organic acids bearing carboxyl/hydroxyl groups—such as acetic, propionic, and citric acids—serve as eco-friendly ligands that coordinate metal ions and enable delayed/controlled crosslinking [104]. As shown in Figure 3, their functional groups underpin chelation in gel networks. Notably, citric acid, due to its tri-carboxylic configuration, affords higher complex stability, promotes the reduction of Cr(Ⅵ) into the less toxic Cr(III), and suppresses the leaching/mobility of chromium species, thereby mitigating ecological risk—consistent with Lockhart’s ligand-exchange theory [105]. Representative coordination strengths and thermal stabilities are summarized in Table 3.

(2) **Multivalent metal substitution.** Replacing toxic chromium with lower-toxicity multivalent cations—e.g., Al(III), Zr(IV), and Ti(IV)—enables controllable gelation and improved thermal endurance. Practical implementations combine delayed-release complexants (e.g., lactate/citrate) with nano-enhancement to sustain long-term integrity under high-temperature/high-salinity conditions. Representative formulations and operating windows are compiled in Table 4.

**Figure 4:**
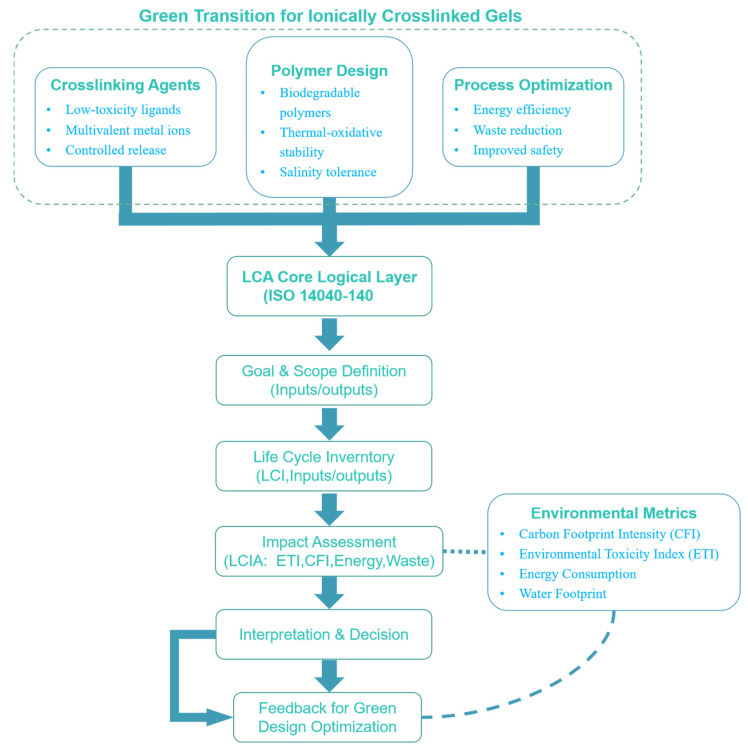
Green transition pathways of metal ion-crosslinked gels and the LCA-based logical evaluation framework.

Overall, these green-substitution strategies substantially mitigate the ecological risks associated with transition-metal gels while maintaining structural robustness, laying the groundwork for sustainable next-generation conformance-control materials. Nevertheless, system-level gaps persist in gelation precision, service durability, and long-term environmental behavior. To systematically quantify these trade-offs, this study employs a life cycle assessment (LCA) framework (Figure 4) that defines explicit system boundaries to evaluate the carbon footprint and environmental burdens throughout synthesis, service, and end-of-life stages.

To quantitatively evaluate these trade-offs, recent studies have introduced life cycle assessment (LCA) approaches to assess the carbon footprint and environmental burdens of metal-ion gels across their synthesis, service, and end-of-life stages [134,135,136,137,138]. Building upon this foundation, we establish an LCA framework [139,140] with clearly defined system boundaries (Figure 4) to examine whether molecular structure tuning and crosslinking-pathway modification can effectively reduce life cycle carbon emissions and ecological burdens. Within this framework, green alternative systems are treated as candidate solutions, offering potential “dual benefits”—network enhancement and toxicity mitigation—though their industrial scalability still depends on high-throughput formulation screening and interfacial-regulation optimization.

A methodological limitation of this analysis is the reliance on globally parameterized LCIA characterization factors; as region-specific datasets (e.g., the emerging Canada-context LCIA) mature [141], recalculation may refine both the absolute impact magnitudes and the relative ranking of alternatives.

#### 2.1.2. Evolution and Green Transition of Organic Gels

As a complement to metal-based gels, organically crosslinked gels have attracted increasing attention due to their renewable raw materials, low toxicity, and tunable crosslinking mechanisms. Current green strategies mainly rely on natural polysaccharides and chitosan to construct metal-free and biodegradable gel networks. These systems offer distinct advantages in terms of gelation precision and ecological safety, and have been successfully applied in pilot-scale oilfield operations [142,143,144].

**Figure 5:**
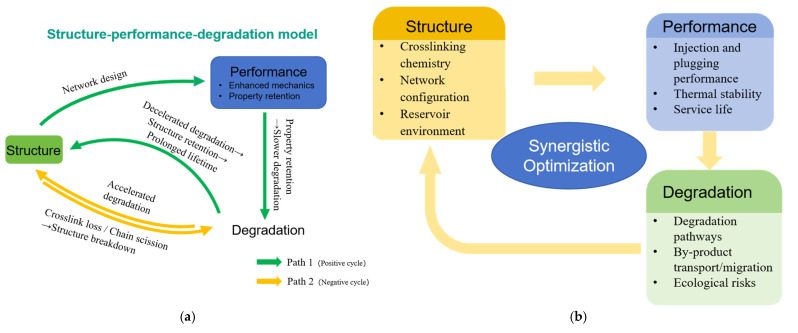
Structure–property–degradation models of organic gels. (**a**) Feedback loop model illustrating the interplay between structure, performance, and degradation; (**b**) coupled optimization model highlighting the balance between performance and degradability.

However, significant challenges remain in achieving a balance between degradability, structural stability, and service performance, which limits their applicability under harsh reservoir conditions such as high temperature, high salinity, and high pressure [145,146,147]. Most studies still emphasize the enhancement of a single property, while lacking a systematic understanding of the synergies between the “crosslinking chemistry–network architecture–reservoir-coupled environment”. This mismatch between design parameters and in situ service conditions hinders large-scale deployment. The key issues can be summarized as follows:

(1) Deviation between “green” labels and actual environmental behavior. Current green evaluations of organic gels primarily emphasize “metal-free” or “low-toxicity” labeling while overlooking their real environmental behavior under subsurface conditions. Most degradation assessments are performed under ambient temperature and neutral pH, failing to capture the actual degradation pathways, migration of byproducts, and ecological risks that occur under high-temperature and high-salinity environments. For instance, tannic acid-based crosslinkers, though commonly regarded as eco-friendly, may generate phenolic intermediates during degradation, potentially leading to groundwater contamination and secondary environmental hazards [148,149].
(2) Trade-off between performance and degradability. Flexible organic gels possess excellent hydrophilicity and biocompatibility but often suffer from insufficient mechanical strength, brittleness, and short service lifetimes under high-temperature and high-pressure conditions [150,151]. For instance, polysaccharide-based gels generally exhibit a loose three-dimensional network and limited toughness due to their high water content [152]. Enhancing thermal stability and plugging efficiency typically requires an increase in crosslinking density or the incorporation of rigid monomers. Although these strategies significantly improve mechanical robustness, they inevitably compromise biodegradability. Conversely, excessive network softening enhances degradability but reduces mechanical endurance, resulting in a clear mismatch between gel strength and degradability (Figure 5a) [153].
(3) Lack of a “structure–property–environment” model. Current design approaches for organic gels remain largely empirical, lacking predictive frameworks capable of describing the coupling effects among molecular structure, performance, and environmental fate. For example, polyethyleneimine (PEI)-based systems can mitigate heavy-metal contamination; however, their long-term environmental behavior and potential toxicological impacts under reservoir conditions remain poorly understood [154,155,156]. This theoretical gap reflects the absence of an integrated understanding of the “green structure–service performance–environmental adaptability” relationship in the existing literature on organic gels.
(4) Need for a coupled “structure–performance–degradation” model. To achieve both structural reliability and environmental compatibility, it is essential to develop a coupled “structure–performance–degradation behavior” model under realistic reservoir boundary conditions. Such a model would provide molecular-level guidance for rational gel design, enabling dynamic optimization between mechanical strength and degradability, and ultimately advancing the green and sustainable transformation of organic gel systems (Figure 5b).

Achieving the effective transformation from laboratory “green labels” to field-scale “green applications” requires overcoming multi-scale design barriers spanning the molecular, mesoscopic, and macroscopic levels. To this end, a synergistic regulatory framework that integrates molecular structure–service behavior–environmental fate must be established. In recent years, bio-based crosslinkers (e.g., citric acid, chitosan, and alginate) [157,158,159], multifunctional responsive monomers [160,161], and combined physical–chemical crosslinking strategies [162,163,164] have provided diverse routes for advancing the green transition of gels. Particularly under the constraints of balancing “greenness–performance” synergy, organic gels are rapidly evolving toward intelligent responsiveness, self-healing capabilities, and modular network architectures. A critical step in this process is the construction of a structure–environmental response–service evolution coupling model. This model, built upon the microscopic configuration of gel networks and incorporating dynamic response mechanisms with reservoir boundary conditions, aims to enhance structural reversibility and environmental adaptability, thereby enabling the transition from traditional “passive plugging” to “active adaptation”. Specific strategies include the following:

(1) Employing dynamic covalent bonds (e.g., Schiff bases and cleavable ester bonds) in synergy with physical association units (e.g., hydrophobic associations and host–guest recognition) to construct reversible networks, thus improving structural reversibility and service stability [71,165,166,167,168,169];
(2) Integrating multicarboxyl/multihydroxyl ligands with degradable monomers to introduce programmable degradation nodes, enabling precise control over service lifetime and ecological release pathways [170,171,172,173];
(3) Leveraging environmental stimuli such as temperature, pH, salinity, and shear disturbances to trigger network reconstruction or phase transitions, thereby promoting dynamic coupling between material performance and multi-field evolution (seepage–mechanical–chemical).

**Figure 6:**
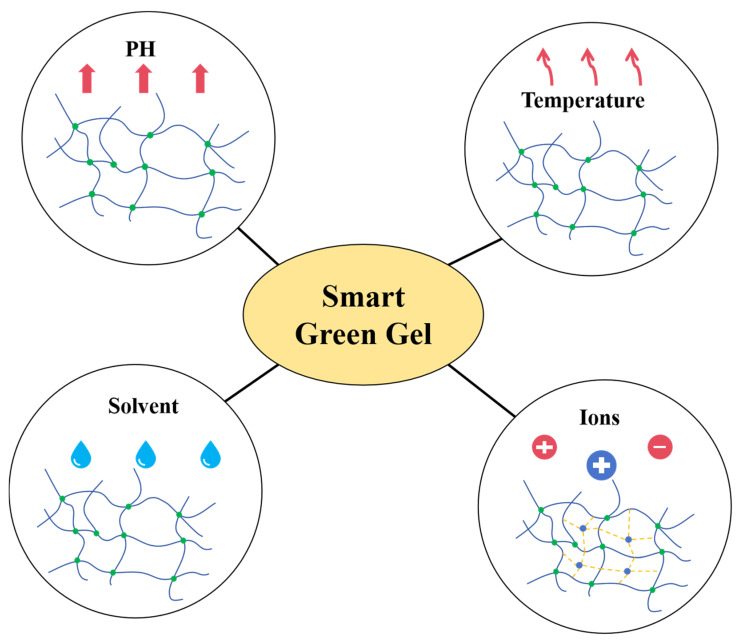
Schematic illustration of the structural adaptation mechanisms of multi-field responsive intelligent gels.

This approach enables full-cycle responsive functions—including timely injection, adaptive plugging, and controlled degradation—demonstrating superior functional stability in complex reservoir conditions (structural response mechanisms illustrated in Figure 6) [174,175,176]. Building upon these advancements, the design of double-network (DN) hydrogels has emerged as an effective pathway for enhancing toughness and service lifetime [177,178,179,180,181]. In such systems, the rigid primary network provides structural support, while the flexible secondary chains dissipate energy and buffer stress, collectively improving strength and deformation resistance [182,183,184,185]. Recent studies utilizing high-throughput simulations have revealed the fracture characteristics and degradation pathways of crosslinking nodes (e.g., citric acid and glutaraldehyde) under extreme reservoir conditions [183], thereby offering theoretical guidance for multi-scale controllable design.

**Figure 7:**
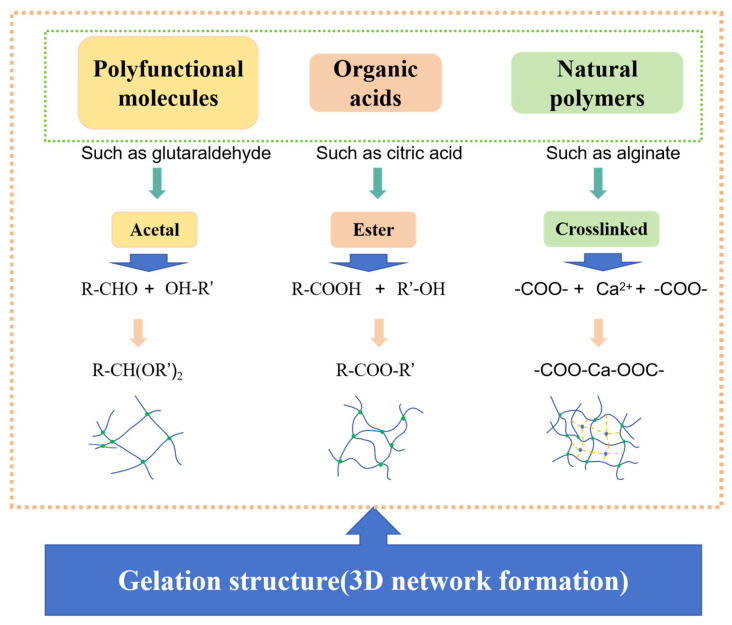
Representative categories of organic gel systems illustrating typical crosslinking reactions and 3D network formation mechanisms [186,187,188,189,190,191].

In summary, the green transformation and intelligent evolution of organic gels are redefining the design paradigm of oilfield profile control materials. Crosslinking pathways differ systematically in precision, stability, and environmental adaptability, yet all face trade-offs between performance and sustainability. Figure 7 and Table 5 highlight the inherent complexity of achieving a “greenness–performance” synergy. Crucially, the long-term stability of these gels in high-temperature, high-salinity, and heterogeneous reservoirs is not solely determined by crosslinking chemistry but also by dynamic evolution under coupled seepage–mechanical–chemical fields. Understanding such failure mechanisms and their geomechanical coupling thus emerges as a central research direction for advancing the functional stability and environmental safety of organic gels.

#### 2.1.3. Profile Control Failure Driven by Reservoir Dynamic Evolution

The pore structure of heterogeneous reservoirs is not static but undergoes continuous evolution under sustained seepage processes [202,203,204,205]. In low-permeability sandstones, for example, the distribution of interstitial materials and grain size gradation determines the advance of the waterflooding front and the sweep efficiency [206]. Studies indicate that pore structure governs seepage behavior through three key mechanisms:

- Pore network topology dictates the connectivity of dominant flow channels [207,208,209,210];
- Particle size distribution controls interfacial tension and capillary resistance [211,212,213,214,215];
- Cementation type affects wettability evolution and displacement sequence [216,217,218,219].

This mechanism has been validated in the Karamay Oilfield, where preferential plugging of macropores induced synergistic imbibition in micropores, thereby enabling a more efficient regulation pathway [220]. Thin-section casting, XRD, and visualized displacement experiments further confirm a strong negative correlation between microscopic heterogeneity and oil recovery efficiency. This provides a systematic recognition chain for intelligent profile control: problem identification (MHI index) → mechanism analysis (graded mobilization) → responsive plugging (material regulation). Table 6 summarizes representative experimental parameters, showing that fluid migration is jointly controlled by pore–throat geometry (throat radius and pore-to-throat volume ratio) [221,222,223,224,225,226] and fluid dynamic parameters (capillary-to-viscous force ratio and pressure gradient, ΔP) [227,228,229,230]. When the permeability contrast, Δk, exceeds 5, profile control agents tend to penetrate high-permeability channels while failing to sufficiently imbibe into low-permeability zones, leading to the phenomenon of “blocking without sealing, sealing without effectiveness” [231,232,233,234]. Further analysis indicates that profile control failure is primarily induced by three categories of multi-field coupling mechanisms:

- Seepage–structure disequilibrium: Pore structure reconstruction facilitates agent escape, reducing plugging efficiency;
- Amplified mechanical disturbance: Injection of blocking agents induces local stress concentration, triggering throat rearrangement, skeleton loosening, or dissolution damage [235,236,237,238];
- Seepage diversion enhances bypass flow, increases injection energy consumption, and necessitates excessive chemical dosage, thereby elevating unit oil-production carbon emissions, aggravating oily wastewater burdens, and increasing the risk of formation dissolution and reservoir integrity loss [239,240,241,242,243].

Experimental results demonstrate that pore-scale architecture and microscopic heterogeneity exert a decisive influence on oil displacement efficiency and waterflood dynamics. Models with lower heterogeneity (X2; X3) achieved higher ultimate recovery factors (30–32%) by improving sweep efficiency, whereas the highly heterogeneous model (X4) exhibited a markedly lower recovery of 20.8% due to rapid breakthrough of dominant channels. Relative wettability indices and water-cut variations further revealed that pore-microstructure coupling not only governs the stability of displacement fronts but also directly dictates the spatial distribution of residual oil. These findings highlight that the effectiveness of profile control gels depends not only on their intrinsic material structure but is also strongly constrained by reservoir heterogeneity and its dynamic evolution. Overall, conventional gels, characterized by structural rigidity and weak adaptability, are prone to failure through channeling, penetration, or dispersion under coupled seepage–mechanical–chemical conditions, reflecting a fundamental contradiction between structural design and dynamic adaptability. Moreover, their lack of precise recognition of dominant channels and tunable blocking capacity results in poor utilization efficiency and limited longevity. Consequently, the development of next-generation green gels with intelligent responsiveness, self-adaptive blocking, and environmental compatibility, along with the establishment of systematic classification and evaluation frameworks, has become a key research direction to drive the green transformation and low-carbon development of profile control technologies.

### 2.2. Green Grading Results

Based on the ETI–CFI framework, four representative gel systems were classified into distinct green performance levels (Table 7).

- Class I (Conventional): Cr(III)-HPAM, exhibiting strong plugging capacity but associated with high toxicity, poor degradability, and a significant carbon footprint.
- Class II (Low-Toxicity): Al-PAM and Zr-PAM, with reduced toxicity compared to Cr-based gels, yet still carbon-intensive.
- Class III (Eco-Friendly): Citric acid–chitosan gels, derived from renewable raw materials, biodegradable, and low in toxicity.
- Class IV (Intelligent Green): pH-responsive nanogels, integrating environmental adaptability, degradability, and functional responsiveness with a low-carbon synthesis pathway.

### 2.3. Case Studies

To validate the applicability of the ETI-CFI green grading framework, four representative gel systems were selected for quantitative comparison. Following the dual-indicator methodology outlined in Table 8 and Table 9, both the Environmental Toxicity Index (ETI) and Carbon Footprint Intensity (CFI) were systematically evaluated. The comparative results are summarized in Table 10. To provide further insight, two systems are presented as illustrative cases.

**Example A. Cr(III)-HPAM Gel (Conventional, Class I)**
**A1. ETI Calculation (weights: toxicity 0.4, persistence 0.2, degradability 0.3, regulation 0.1)**

- Chemical toxicity, *S*_1_ = 0.85: Cr compounds exhibit significant acute and chronic toxicity, while residual acrylamide monomers pose occupational hazards (data source: ECHA/EPA databases);
- Environmental persistence, *S*_2_ = 0.70: Cr is classified as environmentally persistent with bioaccumulation concerns;
- Biodegradability, *S*_3_ = 1.00: HPAM networks are non-biodegradable;
- Regulatory concern, *S*_4_ = 1.00: Cr salts are widely listed as substances of very high concern (SVHC) or subject to strict EPA regulation.

(1) $$ETI=2\times \sum_{i=1}^{4}\left(S_{i}\times W_{i}\right)=2\times (0.85\times 0.4+0.70\times 0.2+1\times 0.3+1\times 0.1)=1.45$$

**A2. CFI Calculation (weights: raw materials 0.5, synthesis 0.2, use 0.2, disposal 0.1)**

- Raw material carbon footprint, $C_{\mathrm{raw}}\;=\;10\;\mathrm{kg}\;{\mathrm{CO}}_{2}\;e\;{kg}^{-1}$: The baseline HPAM footprint ($5–7\;\mathrm{kg}\;{\mathrm{CO}}_{2}\;e/\mathrm{kg}$) was conservatively upscaled to $10\;\mathrm{kg}\;{\mathrm{CO}}_{2}\;e/\mathrm{kg}$ by allocating upstream contributions from Cr-salt production and chelation co-reagents, as well as packaging and transportation. This upscaling was based on existing research and LCA data from reputable sources, including the Ecoinvent and ELCD databases, which provide comprehensive life cycle assessments of similar chemicals and their associated environmental impacts [244,245,246].
- Synthesis energy demand, *S*_2_ = 0.80: Polymerization and post-processing steps are energy-intensive.
- Operational energy demand, *S*_3_ = 0.75: High injection pressure and polymer dosage increase energy input.
- Disposal impact, *S*_4_ = 1.00: Disposal mainly relies on incineration or landfilling with limited valorization pathways.

(2) $$CFI=C_{raw}\times \left[\sum_{j=2}^{4}{(S}_{j}\times W_{j})+W_{1}\right]=10\times \left[(0.80\times 0.2+0.75\times 0.2+1\times 0.1)+0.5\right]=9.1\;kg\;{\mathrm{CO}}_{2}\;e\;{kg}^{-1}$$

**Classification: *ETI* = 1.45, *CFI* = 9.1 ⇒ Class Ⅰ (Conventional).**
**Interpretation**: This system combines effective plugging performance with substantial environmental burdens. The high ETI reflects acute/chronic toxicity, non-biodegradability, and stringent regulatory restrictions. The elevated CFI is driven by raw-material carbon intensity, energy-intensive synthesis, and limited end-of-life valorization. Overall, it constitutes a “performance-oriented but environmentally unsustainable” gel system.
**Example B. Al–PAM/Zr–PAM Gel (Low-Toxicity; Class II)**
**B1. ETI Calculation**

- Chemical toxicity, *S*_1_ = 0.30: Al/Zr salts are far less acutely toxic compared with Cr.
- Environmental persistence, *S*_2_ = 0.20: Low persistence and bioaccumulation concern.
- Biodegradability, *S*_3_ = 0.55: PAM backbone remains largely non-degradable.
- Regulatory concern, *S*_4_ = 0.15: Generally subject to standard regulatory oversight.

(3) $$ETI=2\times \sum_{i=1}^{4}{(S}_{i}\times W_{i})=2\times (0.30\times 0.4+0.20\times 0.2+0.55\times 0.3+0.15\times 0.1)=0.68$$

**B2. CFI Calculation**

- Raw material carbon footprint, $C_{\mathrm{raw}}=5.4\;kg\;{\mathrm{CO}}_{2}\;e\;{kg}^{-1}$: Lower-range PAM values with moderate adjustment for Al/Zr salt preparation.
- Synthesis energy demand, *S*_2_ = 0.60: Partly requires high-temperature and aqueous processing.
- Operational energy demand, *S*_3_ = 0.60: Moderate injection pressure and dosage.
- Disposal impact, *S*_4_ = 0.50: Mainly landfilling with partial valorization options.

(4) $$CFI=C_{raw}\times \left[\sum_{j=2}^{4}{(S}_{j}\times W_{j})+W_{1}\right]=5.4\times \left[(0.60\times 0.2+0.60\times 0.2+0.50\times 0.1)+0.5\right]=4.3\;kg\;{\mathrm{CO}}_{2}\;e\;{kg}^{-1}$$

**Classification: *ETI* = 0.68, *CFI* = 4.3 ⇒ Class II (Low-Toxicity System).**
**Interpretation**: This system demonstrates reduced toxicological risks compared with Cr-based gels, as reflected in its lower ETI score. The substitution of Al/Zr crosslinkers decreases acute toxicity and ecological persistence, yet the use of a non-biodegradable PAM backbone remains a limiting factor. The moderate CFI arises from raw-material requirements, partially energy-intensive synthesis, and disposal routes dominated by landfilling. Overall, it represents a “toxicity-mitigated but still carbon-intensive” gel system, suitable as a transitional alternative but not fully aligned with long-term sustainability targets.
**Example C. Citric Acid-Chitosan Gel (Eco-Friendly, Class III)**
**C1. ETI Calculation**

- Chemical toxicity, *S*_1_ = 0.20: Citric acid and chitosan are considered non-toxic with low occupational exposure risks.
- Environmental persistence, *S*_2_ = 0.10: Not classified as persistent or bioaccumulative.
- Biodegradability, *S*_3_ = 0.10: Chitosan is biodegradable, and the crosslinked network retains degradability.
- Regulatory concern, *S*_4_ = 0.10: Neither component is listed under SVHC or EPA high-priority categories.

(5) $$ETI=2\times \sum_{i=1}^{4}\left(S_{i}\times W_{i}\right)=2\times (0.20\times 0.4+0.10\times 0.2+0.10\times 0.3+0.1\times 0.1)=0.42$$

**C2. CFI Calculation**

- Raw material carbon footprint, $C_{\mathrm{raw}}=2.2\;kg\;{\mathrm{CO}}_{2}\;e\;{kg}^{-1}$: Bio-based chitosan and citric acid; mid-range values from LCA databases and the literature.
- Synthesis energy demand, *S*_2_ = 0.20: Gel formation occurs in mild aqueous conditions at room temperature.
- Operational energy demand, *S*_3_ = 0.30: Relatively low injection pressure and polymer concentration.
- Disposal impact, *S*_4_ = 0.10: Biodegradable with potential for resource recovery.

(6) $$CFI=C_{raw}\times \left[\sum_{j=2}^{4}{(S}_{j}\times W_{j})+W_{1}\right]=2.2\times \left[(0.20\times 0.2+0.30\times 0.2+0.10\times 0.1)+0.5\right]=2.1\;kg\;{\mathrm{CO}}_{2}\;e\;{kg}^{-1}$$

**Classification: ETI = 0.42, CFI = 2.1 ⇒ Class III (Eco-Friendly).**
**Interpretation**: This system exemplifies the benefits of bio-based feedstocks and degradable crosslinkers. The low ETI reflects the non-toxic nature and biodegradability of citric acid and chitosan, as well as their exclusion from major regulatory concern lists. The moderate CFI results from renewable raw materials, mild aqueous synthesis under ambient conditions, relatively low operational energy demands, and environmentally benign disposal pathways. Overall, it constitutes a “biodegradable and carbon-mitigated” gel system, highlighting its strong potential for sustainable oilfield applications.
**Example D. pH-responsive nanogels (Intelligent green; Class IV)**
**D1. ETI Calculation**

- Chemical toxicity, *S*_1_ = 0.10: Derived from natural or bio-based monomers, inherently low toxicity.
- Environmental persistence, *S*_2_ = 0.10: Low persistence with reversible hydration/dehydration.
- Biodegradability, *S*_3_ = 0.20: Introduction of hydrolysable/cleavable linkages enables partial degradability.
- Regulatory concern, *S*_4_ = 0.05: No inclusion of SVHC or high-priority substances.

(7) $$ETI=2\times \sum_{i=1}^{4}\left(S_{i}\times W_{i}\right)=2\times (0.10\times 0.4+0.10\times 0.2+0.20\times 0.3+0.05\times 0.1)=0.25$$

**D2. CFI Calculation**

- Raw material carbon footprint, $C_{\mathrm{raw}}=2.0\;kg\;{\mathrm{CO}}_{2}\;e\;{kg}^{-1}$: Bio-based feedstocks or low-carbon synthetic precursors.
- Synthesis energy demand, *S*_2_ = 0.15: Typically prepared under mild aqueous conditions.
- Operational energy demand, *S*_3_ = 0.25: Low concentration and injection pressure, with potential for self-adaptive swelling/plugging.
- Disposal impact, *S*_4_ = 0.10: Biodegradable with potential for recycling or valorization.

(8) $$CFI=C_{raw}\times \left[\sum_{j=2}^{4}{(S}_{j}\times W_{j})+W_{1}\right]=2.2\times \left[(0.15\times 0.2+0.25\times 0.2+0.10\times 0.1)+0.5\right]\approx 1.2\;kg\;{\mathrm{CO}}_{2}\;e\;{kg}^{-1}$$

**Classification: *ETI* = 0.25, *CFI* = 1.2 ⇒ Class IV (Intelligent Green System)**
**Interpretation**: This system integrates environmental friendliness with functional responsiveness. The very low ETI reflects the use of benign, bio-derived precursors and the absence of major toxicological or regulatory concerns. The low CFI is due to natural raw materials, mild synthesis conditions, minimal injection energy requirements, and fully degradable or recyclable end-of-life pathways. In addition, the pH-responsive network provides controllable plugging and adaptive behavior under reservoir conditions. Overall, it represents a “functionally adaptive and environmentally sustainable” gel system, aligning closely with long-term low-carbon and green development goals.

### 2.4. Framework Validation

The case studies demonstrate that the ETI–CFI framework can systematically differentiate polymer gels; beyond this, it also reveals underlying coupling laws between material properties and environmental performance. First, toxicity and carbon intensity are often correlated but not strictly proportional, as reflected in Al/Zr–PAM systems, which exhibit moderate ETI values but relatively high CFI due to energy- and carbon-intensive synthesis. Second, degradability provides dual benefits by reducing both ETI scores, through lower ecological persistence, and CFI scores, through reduced disposal-related carbon load. Third, intelligent responsiveness enhances adaptability under harsh reservoir conditions while simultaneously lowering operational energy demand, thereby decreasing both indicators.

Collectively, these findings validate the ETI–CFI framework not merely as a classification tool but also as a method capable of capturing performance–environment trade-offs and synergy effects. This methodological robustness strengthens the connection between laboratory-scale evaluation and life cycle environmental assessment, providing a reliable foundation for guiding further research and application.

### 2.5. Engineering and Academic Implications

The ETI–CFI dual-indicator framework not only validates the differentiation of polymer gels across toxicity and carbon intensity dimensions but also provides practical implications for both engineering practice and academic research. From an engineering perspective, it offers a quantitative tool to screen and select green gels prior to field deployment, ensuring that candidate systems satisfy both performance requirements and environmental constraints. This approach reduces trial-and-error in pilot tests, optimizes injection strategies, and minimizes the ecological footprint of chemical profile control operations. From an academic standpoint, the framework establishes a standardized methodology that links molecular-level design with life cycle environmental performance, thereby supporting the rational development of low-toxicity ligands, renewable feedstocks, and degradable polymer networks. Moreover, the construction of open-access ETI–CFI databases can facilitate cross-comparison among gel systems, provide benchmark data for multi-scale modeling, and serve as a foundation for AI-assisted predictive platforms.

To summarize, the proposed framework carries four key implications:

- Quantitative benchmark: Establishes a standardized and transparent metric for evaluating environmental performance across gel systems.
- Design guideline: Provides a systematic reference for reconciling plugging efficiency and structural stability with degradability and environmental compatibility.
- Screening tool: Serves as a practical method for identifying suitable gel systems under dual-carbon policy constraints and environmental regulations.
- Theoretical foundation: Links material innovation with low-carbon development strategies, offering a scientific basis for sustainable substitution and performance optimization in profile control applications.

Collectively, these implications underscore the framework’s value not only as a scientific evaluation tool but also as a strategic guide for advancing polymer gels toward intelligent, low-carbon, and environmentally sustainable oilfield applications.

## 3. Conclusions

This study established and validated a dual-indicator framework combining the Environmental Toxicity Index (ETI) and Carbon Footprint Intensity (CFI) for quantitative evaluation of polymer gels. The results show that Cr(III)-HPAM, while effective in plugging, bears the heaviest environmental burden; citric acid–chitosan systems markedly reduce toxicity and carbon emissions; and pH-responsive gels achieve a balance between stability and sustainability. Key challenges remain, including reconciling strength with degradability, accounting for dynamic reservoir conditions, and addressing the lack of open ETI–CFI databases. Future work should emphasize multi-scale modeling, reconfigurable gel networks, and AI-assisted predictive platforms to accelerate the transition toward intelligent, low-carbon, and environmentally sustainable oilfield applications.

## 4. Materials and Methods

This section details the proposed ETI–CFI framework, including its theoretical basis and calculation logic for evaluating the environmental performance of polymer gels.

### 4.1. Green Grading Framework

**Figure 8:**
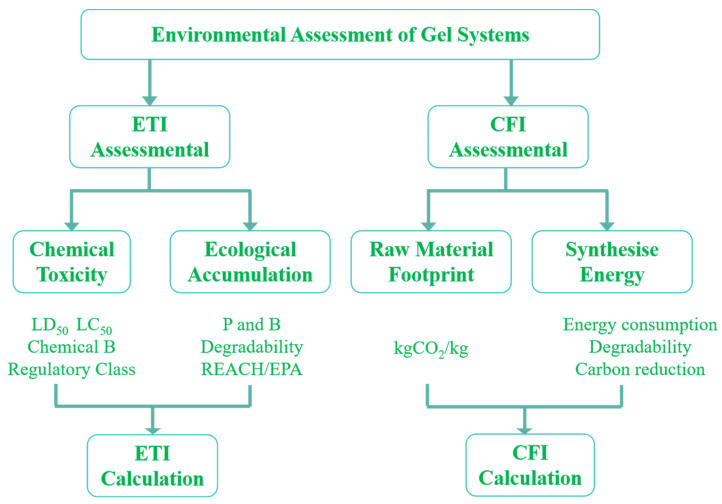
Flowchart of environmental friendliness evaluation.

The classification of polymer gels was developed using a dual-indicator framework that integrates the Environmental Toxicity Index (ETI) and Carbon Footprint Intensity (CFI). This framework enables systematic comparison and hierarchical classification of gel systems into four categories—conventional, low-toxicity, eco-friendly, and intelligent green—based on toxicological attributes, life cycle carbon emissions, degradability, and environmental adaptability (Figure 8). It provides a quantitative basis for assessing green material performance and guiding sustainable formulation design.

**Data Sources and Theoretical Basis** [247,248,249,250]

The classification criteria and corresponding threshold ranges presented in Table 8 were not derived from new experimental measurements but were formulated through a systematic integration and critical analysis of existing research, publicly available databases, and international regulatory documents. This approach ensures that the proposed evaluation system is grounded in established environmental benchmarks and consistent with international sustainability assessment standards. Specifically, the toxicological indicators were sourced from the European Union’s REACH Substances of Very High Concern (SVHC) list and the U.S. Environmental Protection Agency (EPA) Priority Substance database, while the carbon footprint boundaries were determined in accordance with life cycle assessment (LCA) standards such as ISO 14067 and the PlasticsEurope eco-profile database. Together, these datasets provide a coherent and comparable foundation for constructing a quantitative scoring framework to evaluate the sustainability of polymer gels based on consolidated knowledge from previous studies.

**Evaluation Procedure**

As shown in Figure 8, the evaluation process consists of three sequential stages:

- **Toxicological and Regulatory Screening (ETI):** The intrinsic environmental hazards of gel components are assessed through acute and chronic toxicity (e.g., LD_50_, LC_50_, NOEC), ecological persistence and bioaccumulation, biodegradability, and regulatory concern (e.g., inclusion in REACH SVHC or EPA priority lists). These indicators are aggregated to yield the ETI, which reflects the overall toxicological risk.
- **Carbon Footprint Accounting (CFI):** Using life cycle assessment (LCA), greenhouse gas emissions associated with raw material acquisition, synthesis, application, and end-of-life treatment are quantified. The results are expressed as standardized emissions per unit mass of gel (kg CO_2_e kg^−1^), thereby capturing both material-specific burdens and process-related energy inputs.
- **Comprehensive Grading and Classification:** ETI and CFI values are jointly mapped onto a dual-axis evaluation framework. This enables systematic comparison of different gel systems and classification into distinct green performance levels, ranging from traditional heavy-metal gels to organic crosslinked systems, bio-based degradable gels, and intelligent green gels.

**Threshold Definition and Classification Criteria**

The ETI–CFI ranges listed in Table 8 were determined through comparative analysis of representative gel systems, integrating toxicological and carbon-emission data from the aforementioned sources. ETI values were normalized between 0 and 2 according to toxicological weightings, while CFI values were derived from standardized CO_2_-equivalent emissions per unit mass of gel.

The threshold intervals correspond to the quartile distribution of existing polymer gel datasets, reflecting a progressive enhancement in environmental performance—from conventional Cr(III)–PAM gels (ETI > 1.2; CFI > 8) to intelligent green gels (ETI < 0.3; CFI < 1.5).

This quantitative–qualitative coupling enables the ETI–CFI framework to serve as a unified criterion for evaluating the sustainability of polymer gel systems in oilfield applications.

### 4.2. Environmental Friendliness Evaluation

#### 4.2.1. Calculation Logic of Environmental Toxicity Index (ETI)

The ETI quantifies the environmental risk of gel systems by integrating four dimensions:

- Chemical toxicity: Acute and chronic toxicity indicators such as LD50/LC50 and NOEC, typically obtained from authoritative databases (e.g., ECHA, EPA) [251,252,253,254];
- Ecological persistence and bioaccumulation: Classification of persistent (P), bioaccumulative (B), very persistent (vP), or very bioaccumulative (vB) substances [255,256,257,258,259];
- Degradability: Chemical and biological degradability, based on standardized OECD 301B tests (e.g., chitosan is degradable, while HPAM is non-degradable);
- Regulatory concern: whether the material is listed as a Substance of Very High Concern (SVHC) under REACH or on the EPA priority control list [260,261,262,263].

Each indicator was normalized to a 0–1 scale and assigned weights of 0.4, 0.2, 0.3, and 0.1, respectively. The final ETI value was calculated through weighted summation and normalized to the 0–2 range to enhance differentiation. A higher ETI indicates stronger environmental toxicity, and the detailed scoring criteria are summarized in Table 9. The calculation formula is as follows:

(9) $$ETI=2\times \sum_{i=1}^{4}\left(S_{i}\times W_{i}\right),$$

where $S_{i}$ is the normalized score of the i-th indicator, and $W_{i}$ is the assigned weight. A multiplication factor of 2 is introduced to expand the ETI value into the 0–2 range, thereby improving differentiation between materials of varying environmental risks.

For example, Cr(III)-HPAM gels exhibit high toxicity and poor degradability, contain heavy metals, and are listed on regulatory concern lists—thus yielding a relatively high score. In contrast, citric acid–chitosan gels originate from natural sources, are low in toxicity, biodegradable, and free from regulatory restrictions—resulting in a relatively low score. Overall, a higher ETI value indicates stronger environmental toxicity.

**a. Normalization and Weight Determination.**

All indicators in the ETI framework were normalized using a min–max linear transformation to a 0–1 scale according to Equation (10):

(10) $$S_{i}=\frac{X_{i}-X_{min}}{X_{max}-X_{min}}$$

where $X_{i}$ represents the measured or reported value of each indicator, and $X_{min}/X_{max}$ denote the minimum and maximum reference values obtained from authoritative databases such as ECHA and EPA. The selection of these boundary values followed the recommended ranges for acute toxicity (LD_50_/LC_50_), degradability, persistence, and bioaccumulation parameters provided in regulatory or OECD testing guidelines. Higher toxicity, lower degradability, or greater persistence correspond to higher normalized scores.

The weighting factors (Wi) were determined through a modified Delphi approach that evaluates the relative importance of each environmental dimension, using a modified Delphi approach and supported by previous LCA-based ecotoxicity weighting studies [251].

The Delphi process consisted of two iterative consultation rounds involving five experts in polymer chemistry, environmental toxicology, and petroleum engineering. In each round, experts independently ranked the importance of toxicity, persistence/bioaccumulation, degradability, and regulatory concern on a 0–10 scale. The mean and interquartile range (IQR) of expert scores were analyzed to assess consensus; indicators with an IQR ≤ 2 were considered stable. Final weights were obtained by averaging the second-round consensus values and normalizing their sum to unity, resulting in the final weighting vector (0.4, 0.2, 0.3, 0.1).

To ensure future reproducibility and facilitate large-scale application, the weighting matrix ($W_{i}$) and normalized indicator scores ($S_{i}$) can be systematically parameterized as input variables for a dedicated ETI–CFI computational module. The normalization standards for each indicator are derived from regulatory and database-defined reference ranges—specifically, OECD testing guidelines and values reported in ECHA and EPA datasets—ensuring consistent interpretation of toxicity, degradability, and persistence indicators across different materials. The weighting scheme is determined using the expert-consensus results obtained from the modified Delphi process, in which the mean and IQR of expert ratings define the relative contribution of each dimension to the overall ETI value. This module can be developed in Python version 3.9.24, Excel, or MATLAB R2020a environments to automatically compute ETI and CFI values through matrix operations based on Equations (9) and (10). The proposed algorithm would read raw indicator data, perform min–max normalization, apply expert-derived weights, and output aggregated scores for each gel system. Such a digital implementation framework provides a transparent and standardized calculation procedure, ensuring reproducibility of normalization and weighting across studies. Moreover, it supports sensitivity and Monte Carlo simulations to quantify uncertainty propagation within the ETI–CFI evaluation process and lays the foundation for integration into future decision-support or life cycle assessment (LCA) software platforms for green-material evaluation. In summary, these procedures establish explicit standards for the selection and calculation of normalized indicator scores and weighting factors in the ETI framework, ensuring methodological transparency and reproducibility.

#### 4.2.2. Carbon Footprint Intensity (CFI) Calculation Logic

The Carbon Footprint Intensity (CFI) quantifies the greenhouse gas emissions associated with the full life cycle of polymer gels. The evaluation includes four stages:

- Raw material footprint (kg CO_2_e kg^−1^): Data are obtained from established life cycle assessment (LCA) databases—such as Ecoinvent (https://ecoinvent.org), GaBi LCA (https://sphera.com/gabi/, accessed on 11 October 2025), and ELCD (https://eplca.jrc.ec.europa.eu/, accessed on 11 October 2025)—or derived from reports from the literature. For reference, typical values include HPAM ≈ 5–7 kg CO_2_e kg^−1^ and chitosan ≈ 2 kg CO_2_e kg^−1^ [264].
- Synthesis energy consumption: Determined by whether high-temperature/high-pressure reactions are required (e.g., HPAM synthesis involves energy-intensive conditions, while chitosan–citric acid gels can be formed under ambient conditions).
- Operational energy consumption: Accounts for injection pressure and concentration requirements during field application (polymer-based systems typically require higher injection pressure).
- End-of-life treatment: Considers degradability and potential for resource recovery (e.g., chitosan gels are biodegradable, whereas Cr-based gels present significant disposal challenges).

Except for the raw material footprint (Stage 1), which is obtained from experimental measurements or LCA databases, the remaining stages (Stages 2–4) are normalized to a 0–1 scale to capture relative differences. The assigned weights for the four stages are 0.5, 0.2, 0.2, and 0.1, respectively. A higher CFI indicates greater carbon emission intensity, and the detailed scoring framework is summarized in Table 10. The final calculation formula is expressed as follows:

(11) $$CFI=C_{raw}\times \left[\sum_{j=2}^{4}{(S}_{j}\times W_{j})+W_{1}\right],$$

where $C_{raw}$ represents the raw material carbon footprint (kg CO_2_e kg^−1^) obtained from LCA databases, $S_{j}$ denotes the stage-specific scores, and $W_{j}$ indicates the assigned weight for each stage.

For example, the Cr(III)–HPAM system exhibits a high carbon footprint due to large raw material emissions, high synthesis energy demand, significant injection pressure, and disposal dominated by incineration or landfilling, leading to a high CFI value. In contrast, pH-responsive gels are based on natural raw materials, synthesized under ambient conditions, and partially biodegradable, thus resulting in a relatively low CFI value. In general, a higher CFI corresponds to greater carbon emission intensity throughout the gel life cycle.

**b. Normalization and Data Sources.**

For CFI evaluation, life cycle data for each stage were collected from Ecoinvent v3.10, GaBi 2023, ELCD 3.2, and peer-reviewed literature. Non-dimensional scores ($S_{j}$) were derived using min–max normalization across representative gel systems (HPAM, chitosan, alginate, polysaccharide derivatives). Weighting factors ($W_{j}$) were optimized through sensitivity analysis, ensuring that the dominant contributor—raw material footprint—accounts for approximately 50% of total CFI, consistent with LCA best practices. The normalized and weighted data were then aggregated according to Equation (10) to obtain the composite CFI. Accordingly, the adopted normalization and weighting criteria establish unified and transparent standards for the selection and calculation of all indicators within the CFI framework, ensuring comparability and reproducibility across different polymer gel systems.

## Figures and Tables

**Table 1:** Environmentally oriented classification and performance of polymer gels [39].

Type	Definition and Characteristics	Representative Raw Materials/Crosslinkers	Environmental Performance and Challenges
Conventional (non-green)	Synthetic polymer networks crosslinked with Cr(III) or aldehydes; high stability but poor degradability [40,41,42].	PAM with chromium/zirconium salts, glutaraldehyde.	High Cr(Ⅵ) toxicity, VOCs, and microplastic pollution; should be phased out.
Low-toxicity/low-pollution	Formulations using low-toxicity crosslinkers to reduce VOCs and improve polymer compatibility [43,44,45,46].	Citric acid, aluminum salts, organic titanium.	Lower toxicity but limited durability and aging resistance.
Green eco-friendly	Renewable polymer matrices with biodegradable crosslinkers; naturally degradable after service [47,48,49,50,51].	Alginate, chitosan, cellulose; citric or oxalic acids.	Excellent biodegradability but low salt tolerance and mechanical strength.
Intelligent green	Responsive gels with thermo-/pH-/shear-adaptive networks for targeted plugging [52,53,54].	Responsive polysaccharides or peptide-based crosslinkers.	Good adaptability; complex synthesis and high cost.

**Table 2:** Representative formulations and performance of transition-metal gels under different reinforcement strategies.

Reinforcement Strategy	Key Mechanism/Technique	Representative Formulation	Applicable T/Environment	Application Scenario	Reference
Host–guest inclusion	β-Cyclodextrin–adamantane supramolecular interaction	PAAB: PAMN = 1:1	90 °C; salinity ≈ 32,900 mg L^−1^	High-T, high-salinity reservoirs (EOR); drug delivery models	Zhou [91]
Thermo-responsive host–guest inclusion	Temperature-sensitive β-CD/Ad host–guest interaction; intermolecular binding energy analysis	β-CD/Ad complex	Stable at 330–340 K (57–67 °C)	Smart delivery systems; potential for thermo-responsive gels in EOR	Rasouli [92]
Zwitterionic hydrophobic monomer design	Sulfonic, phenyl, and long-alkyl monomer incorporation to enhance rigidity and salt tolerance	CQMP(AM/SSS/CQ)	Stable > 130 °C; decomposition ≈ 210 °C	High-T, high-salinity reservoirs (EOR)	Cheng [93]
Nano-fly ash composite	Nano-FA improves thermal stability and strength	AM/nano-FA/Cr(III) system	90 °C; ΔP = 0.045 MPa; ηplug = 95.1%	Fractured reservoirs (water control)	Singh [94]
Zwitterionic copolymer reinforcement	Charge neutralization and ion-shielding compensation improve salinity tolerance and hydration stability	Poly(sulfobetaine-co-acrylamide) (P(SB-co-AM)); Poly(carboxybetaine-co-AMPS)	High salinity (>200,000 mg L^−1^ NaCl), 90–120 °C	High-salinity reservoirs; salt-resistant rheology modifiers	Lowe and McCormick [95]
Pickering emulsion flooding	Amphiphilic copolymer-stabilized anionic emulsion	AMPSA/AA/DMA	94% displacement efficiency	Residual-oil recovery in porous media	Ntente [96]
Supramolecular dynamic network	Hydrophobic and H-bond crosslinking via methacrylate–ammonium system.	Methacrylate/DTAB	135 °C; compressive strength ≈ 14.5 MPa	Cavernous/deep reservoirs	Yang [97]
Active amphiphilic polymer	C_16_ long-chain hydrophobic association.	AM/AA/C16DMAAC	>95% viscosity reduction at 60 °C	Heavy oil reservoirs (EOR)	Yang [98]
Al_2_O_3_ nanoparticle filling	Nanoparticles improve high-T/salinity tolerance and thermal stability	NaSS/DMA/Al_2_O_3_/Cr(III)	Stable 300 days at 150 °C	High-T aging reservoirs (plugging)	Pandit [99]

**Table 3:** Representative organic ligands and their corresponding performance parameters.

Organic Ligand	Structural Features	Chelation Ability	Release Control Ability	Thermal Stability	Representative References
Acetic acid	Monocarboxylic acid; limited coordination sites.	Medium	Medium	Medium	Guanghua Yi and Michael Sayer [106,107], Kang [108]
Propionic acid	Longer chain; slightly stronger chelation.	Medium	Medium	Medium	Mumallah [109], Kaddouri [110]
Boric acid	Trihydroxy structure; stable borate complexation.	Strong	Strong	Strong	Citeseer [111], Shah et al. [112], Brannon [113], Harris [114]
Lactic acid	α-Hydroxy and carboxyl groups; stabilizes Cr(III) complexes.	Strong	Relatively strong	Strong	Yang et al. [82], Jong et al. [115]
Citric acid	Tricarboxylic; multi-site coordination with hydroxyl groups.	Strong	Strong	Strong	Niu et al. [116], Takahashi [117]
Phenolic resin	Polyhydroxy aromatic; forms rigid chelated networks.	Strong	Relatively strong	Relatively strong	Shibayama [118], Ran et al. [119]

**Table 4:** Representative enhancement strategies, formulations, and application conditions.

Enhancement Strategy	Key Technique/Mechanism	Representative Formulation	Applicable Conditions	Performance Advantages
Hydrophobic association [120,121,122]	Styrene/acrylate monomers form hydrophobic domains	HPAM-St	100–140 °C, high salinity	Improved elasticity and shear resistance.
Host–guest inclusion [123,124,125]	β-CD–adamantane supramolecular complexation	βCD-g-PAM	>90 °C, salinity 1.5 × 10^5^ mg L^−1^	Controllable density, low-dosage efficiency.
Nanofiller incorporation [126,127,128]	SiO_2_, montmorillonite, or fly ash reinforcement	HPAM/SiO_2_	Up to 160 °C	Enhanced thermal strength and plugging.
Organic chelation [129,130,131]	Citric acid–Cr(III) coordination	HPAM-Cr(III)-citric acid	Delayed gelation (24–480 h)	Tunable gelation, reduced toxicity.
Metal substitution [132,133]	Ti(Ⅵ), Al(III), Fe(III) replacing Cr(III)	HPAM–Ti(IV)	Delayed gelation (24–480 h)	Tunable gelation; reduced toxicity

**Table 5:** Comparison of crosslinking mechanisms, technical features, and limitations of three representative organic gel systems.

System Type	Cross Linking Mechanism	Technical Features	Limitations
Small-molecule aldehydes [192,193,194]	Schiff base condensation (–C=N–)	Adjustable gelation time (10–300 min)	Formaldehyde/glutaraldehyde residues (LC_50_ = 12 mg L^−1^)
Organic acid esterification [195,196,197,198,199]	Carboxylic ester bonding (–COO–)	pH responsiveness (swelling ratio > 300%)	Ester hydrolysis under high salinity (modulus loss > 60%)
Natural polymer ion-bridging [200,201]	Divalent cation coordination [Ca(II)/Mg(II)]	High biodegradability (>80%, OECD 301B)	Network dissociation at high temperature (>80 °C)

**Table 6:** Experimental data on the correlation between reservoir pore–microscopic heterogeneity parameters and multi-stage oil displacement efficiency under water flooding dynamics [206].

Model	Pore-Scale Heterogeneity	Microscopic Heterogeneity	Oil Recovery (Water-Free Stage, %)	Final Oil Recovery (%)	Relative Wettability Index	Water-Cut Variation Rate
X1	High	Low	6.3	25	0.17	3.5
X2	Low	High	10.9	31.8	0.47	2.7
X3	Lowest	Lowest	8.5	30.8	0.35	3.8
X4	Highest	Highest	5.0	20.8	0.51	2.9
X5	Medium	Medium	6.9	25	0.18	2.8

**Table 7:** Quantitative comparison of green performance of different types of polymer gels.

Type	Representative System	ETI Score	CFI Score (kgCO_2_e/kg)	Classification Validation
Class Ⅰ Conventional	Cr(III)-PAM	1.45	9.1	Meets Class Ⅰ criteria
Class II Low-toxicity	Al-PAM/Zr-PAM	0.68	4.3	Meets Class II criteria
Class III Eco-friendly	Citric acid–chitosan	0.42	2.1	Meets Class III criteria
Class IV Intelligent green	PH-responsive nanogels	0.25	1.2	Meets Class IV criteria

**Table 8:** Classification criteria for green polymer gels (developed based on REACH, EPA, ISO 14067, and PlasticsEurope databases).

Level	Definition	ETI Value	CFI (kgCO_2_e/kg)	Typical Features	Representative Systems
I. Conventional	High efficiency; high toxicity and carbon footprint.	>1.2	>8	Non-degradable; Cr(III) or aldehyde/phenolic crosslinkers.	Cr(III)-HPAM, glutaraldehyde–PAM gels
II. Low-toxicity	Lower toxicity; moderate carbon intensity.	0.5–0.8	3–8	Partial replacement of toxic ions or organics.	Al–PAM, Zr–HPAM, organic acid–PAM gels
III. Eco-friendly	Renewable and partially degradable networks.	0.3–0.5	1.5–3	Biopolymer matrix; low-toxicity organic crosslinkers.	Citric acid–chitosan, alginate, scleroglucan gels
IV. Intelligent green	Responsive, degradable, low-carbon systems.	<0.3	<1.5	Stimuli-responsive, self-healing, bio-based dual networks.	pH-responsive nanogels, double-network gels

**Table 9:** Evaluation framework for Environmental Toxicity Index (ETI).

Evaluation Dimension	Weight (Suggested)	Scoring Criteria (Example)	Score Range
Chemical Toxicity	0.4	LD_50_/LC_50_ < 50 mg/kg→ high score; higher LD_50_/LC_50_ → lower score	0~1
Ecological Persistence and Bioaccumulation	0.2	Persistent (P) + Bioaccumulative (B) → maximum score; no bioaccumulation → minimum score	0~1
Degradability	0.3	Completely degradable → minimum score; non-degradable → maximum score	0~1
Regulatory Concern	0.1	Listed in REACH SVHC or EPA priority list → maximum score	0~1

**Table 10:** Evaluation framework for Carbon Footprint Intensity (CFI).

Evaluation Dimension	Weight (Suggested)	Scoring Criteria (Example)	Score Range
Raw material carbon footprint	0.5	Based on LCA database values, kg (CO_2_e kg^−1^)	0–1
Energy consumption during synthesis	0.2	High-temperature/high-pressure synthesis = high score; ambient-temperature synthesis = low score	0–1
Energy demand during application	0.2	High injection energy or difficulty = high score	0–1
Post-treatment carbon emissions	0.1	Incineration/landfilling = high score; biodegradable/recyclable = low score	0–1

## Data Availability

All data generated or analyzed during this study are included in this published article.

## Abbreviations

The following abbreviations are used in this manuscript:

ETI	Environmental Toxicity Index
CFI	Carbon Footprint Intensity
HPAM	Hydrolyzed Polyacrylamide
LCA	Life Cycle Assessment
REACH	Registration, Evaluation, Authorisation and Restriction of Chemicals
SVHC	Substances of Very High Concern
OECD	Organisation for Economic Co-operation and Development

## References

[1] Liang B., Chen C., Jia C., Wang C., Wang X., Zha Y., Wang R., Meng Z., Wang H. (2024). Carbon capture, utilization and storage (CCUS) in oil and gas reservoirs in China: Status, opportunities and challenges. Fuel.

[2] Zhao S., Song Q., Liu L., Li J., Zhao D. (2024). Uncovering the lifecycle carbon emissions and its reduction pathways: A case study of petroleum refining enterprise. Energy Convers. Manag..

[3] Shang L., Lyu Z., Sun N., Shen G., Shen Q., Guo R., Wei W. (2025). Pathways for supply security and carbon-neutral transition in the oil products industry: A comprehensive technology portfolio evaluation. J. Clean. Prod..

[4] Zou C., Lin M., Ma F., Liu H., Yang Z., Zhang G., Yang Y., Guan C., Liang Y., Wang Y. (2024). Development, challenges and strategies of natural gas industry under carbon neutral target in China. Pet. Explor. Dev..

[5] Friedlingstein P., O’Sullivan M., Jones M., Andrew R., Bakker D., Hauck J., Landschützer P., Le Quéré C., Luijkx I., Peters G. (2023). Global Carbon Budget 2023. Earth Syst. Sci. Data.

[6] Cointe B., Guillemot H. (2023). A history of the 1.5 °C target. WIREs Clim. Change.

[7] Kuyper J., Schroeder H., Linnér B.-O. (2018). The Evolution of the UNFCCC. Annu. Rev. Environ. Resour..

[8] Schipper E.L.F. (2006). Conceptual History of Adaptation in the UNFCCC Process. Rev. Eur. Community Int. Environ. Law.

[9] IEA (2025). Global Energy Review 2025.

[10] Finkbeiner M., Inaba A., Tan R., Christiansen K., Klüppel H.-J. (2006). The New International Standards for Life Cycle Assessment: ISO 14040 and ISO 14044. Int. J. Life Cycle Assess..

[11] Wu P., Xia B., Wang X. (2015). The contribution of ISO 14067 to the evolution of global greenhouse gas standards—A review. Renew. Sustain. Energy Rev..

[12] Rebitzer G., Ekvall T., Frischknecht R., Hunkeler D., Norris G., Rydberg T., Schmidt W.P., Suh S., Weidema B.P., Pennington D.W. (2004). Life cycle assessment: Part 1: Framework, goal and scope definition, inventory analysis, and applications. Environ. Int..

[13] Schrijvers D., Loubet P., Sonnemann G. (2020). Archetypes of Goal and Scope Definitions for Consistent Allocation in LCA. Sustainability.

[14] Van Hoof G., Vieira M., Gausman M., Weisbrod A. (2013). Indicator selection in life cycle assessment to enable decision making: Issues and solutions. Int. J. Life Cycle Assess..

[15] Goedkoop M., Heijungs R., Huijbregts M., De Schryver A., Struijs J., Van Zelm R. (2009). ReCiPe 2008. A life cycle impact assessment method which comprises harmonised category indicators at the midpoint and the endpoint level. Impact World.

[16] Owsianiak M., Hauschild M.Z., Posthuma L., Saouter E., Vijver M.G., Backhaus T., Douziech M., Schlekat T., Fantke P. (2023). Ecotoxicity characterization of chemicals: Global recommendations and implementation in USEtox. Chemosphere.

[17] Rosenbaum R.K., Bachmann T.M., Gold L.S., Huijbregts M.A.J., Jolliet O., Juraske R., Koehler A., Larsen H.F., MacLeod M., Margni M. (2008). USEtox—The UNEP-SETAC toxicity model: Recommended characterisation factors for human toxicity and freshwater ecotoxicity in life cycle impact assessment. Int. J. Life Cycle Assess..

[18] Henderson A.D., Hauschild M.Z., van de Meent D., Huijbregts M.A.J., Larsen H.F., Margni M., McKone T.E., Payet J., Rosenbaum R.K., Jolliet O. (2011). USEtox fate and ecotoxicity factors for comparative assessment of toxic emissions in life cycle analysis: Sensitivity to key chemical properties. Int. J. Life Cycle Assess..

[19] Rosenbaum R.K., Huijbregts M.A.J., Henderson A.D., Margni M., McKone T.E., van de Meent D., Hauschild M.Z., Shaked S., Li D.S., Gold L.S. (2011). USEtox human exposure and toxicity factors for comparative assessment of toxic emissions in life cycle analysis: Sensitivity to key chemical properties. Int. J. Life Cycle Assess..

[20] Belyanovskaya A., Laratte B., Perry N., Baranovskaya N. (2019). A regional approach for the calculation of characteristic toxicity factors using the USEtox model. Sci. Total Environ..

[21] Bare J.C. (2002). Traci. J. Ind. Ecol..

[22] Bare J. (2011). TRACI 2.0: The tool for the reduction and assessment of chemical and other environmental impacts 2.0. Clean Technol. Environ. Policy.

[23] Sydansk R.D., Southwell G. (2000). More than 12 years’ experience with a successful conformance-control polymer-gel technology. SPE Prod. Fac..

[24] Frampton H., Morgan J.C., Cheung S.K., Munson L., Chang K.T., Williams D. Development of a novel waterflood conformance control system. Proceedings of the SPE/DOE Symposium on Improved Oil Recovery.

[25] Sagbana P.I., Abushaikha A.S. (2021). A comprehensive review of the chemical-based conformance control methods in oil reservoirs. J. Pet. Explor. Prod. Technol..

[26] Imqam A., Bai B., Delshad M. (2018). Micro-particle gel transport performance through unconsolidated sandstone and its blocking to water flow during conformance control treatments. Fuel.

[27] Sheng J.J., Leonhardt B., Azri N. (2015). Status of Polymer-Flooding Technology. J. Can. Pet. Technol..

[28] Adewunmi A.A., Ismail S., Sultan A.S. (2018). Crosslinked Polyacrylamide Composite Hydrogels Impregnated with Fly Ash: Synthesis, Characterization and Their Application as Fractures Sealant for High Water Producing Zones in Oil and Gas Wells. J. Polym. Environ..

[29] Ghosh B., Ali S.A., Belhaj H. (2020). Controlling excess water production in fractured carbonate reservoirs: Chemical zonal protection design. J. Pet. Explor. Prod. Technol..

[30] Song R., Jiang G., Wang K. (2018). Gelation mechanism and rheological properties of polyacrylamide crosslinking with polyethyleneimine and its plugging performance in air-foam displacement. J. Appl. Polym. Sci..

[31] Wang K., Luo M., Li M., Gu X., Li X., Fan Q., Pu C., Wang L. (2024). Gelation and Plugging Performance of Low-Concentration Partially Hydrolyzed Polyacrylamide/Polyethyleneimine System at Moderate Temperature and in Fractured Low-Permeability Reservoir. Polymers.

[32] Niu C., Fan S., Chen X., He Z., Dai L., Wen Z., Li M. (2024). Preparation and Performance Evaluation of a Supramolecular Polymer Gel-Based Temporary Plugging Agent for Heavy Oil Reservoir. Gels.

[33] Sang Q., Li Y., Yu L., Li Z., Dong M. (2014). Enhanced oil recovery by branched-preformed particle gel injection in parallel-sandpack models. Fuel.

[34] Tongwa P., Baojun B. (2015). A more superior preformed particle gel with potential application for conformance control in mature oilfields. J. Pet. Explor. Prod. Technol..

[35] Bai B., Zhou J., Yin M. (2015). A comprehensive review of polyacrylamide polymer gels for conformance control. Pet. Explor. Dev..

[36] Xiong B., Loss R.D., Shields D., Pawlik T., Hochreiter R., Zydney A.L., Kumar M. (2018). Polyacrylamide degradation and its implications in environmental systems. npj Clean Water.

[37] Meng M., Niu D., Shang W. (2012). CO2 emissions and economic development: China’s 12th five-year plan. Energy Policy.

[38] (2020). Technical Evaluation Methods for Environmental Protection of Water-Soluble Oilfield Chemicals.

[39] Lei S., Sun J., Lv K., Zhang Q., Yang J. (2022). Types and Performances of Polymer Gels for Oil-Gas Drilling and Production: A Review. Gels.

[40] Burrafato G., Carminati S., Bonaccorsi F., Lockhart T.P. (1990). Evidence for molecular Cr^3+^ cross-links in Cr^3+^/polyacrylamide gels. Macromolecules.

[41] te Nijenhuis K. (2001). Crosslink nature in Cr(III)-polyacrylamide gels. Macromol. Symp..

[42] Zhang S., Guo J., Gu Y., Zhao Q., Yang R., Yang Y. (2020). Polyacrylamide gel formed by Cr(III) and phenolic resin for water control in high-temperature reservoirs. J. Pet. Sci. Eng..

[43] Shaiful Bahari A.M., Othman S.Z., Mohamad Fadli M.F., Zulkifli M.Z.A., Biyamin S.A., Islam M.A., Aspanut Z., Amin N., Misran H. (2021). Facile synthesis of Zr-based metal-organic gel (Zr-MOG) using “green” sol-gel approach. Surf. Interfaces.

[44] Dietrich D., Licht C., Nuhnen A., Höfert S.-P., De Laporte L., Janiak C. (2019). Metal–Organic Gels Based on a Bisamide Tetracarboxyl Ligand for Carbon Dioxide, Sulfur Dioxide, and Selective Dye Uptake. ACS Appl. Mater. Interfaces.

[45] Zhang H., Yang H., Sarsenbekuly B., Zhang M., Jiang H., Kang W., Aidarova S. (2020). The advances of organic chromium based polymer gels and their application in improved oil recovery. Adv. Colloid Interface Sci..

[46] Liu G., Li S., Shi C., Huo M., Lin Y. (2023). Progress in Research and Application of Metal–Organic Gels: A Review. Nanomaterials.

[47] Nishinari K., Zhang H., Ikeda S. (2000). Hydrocolloid gels of polysaccharides and proteins. Curr. Opin. Colloid Interface Sci..

[48] Clark A.H. (1996). Biopolymer gels. Curr. Opin. Colloid Interface Sci..

[49] Chowhan A., Giri T.K. (2020). Polysaccharide as renewable responsive biopolymer for in situ gel in the delivery of drug through ocular route. Int. J. Biol. Macromol..

[50] Xia S., Zhang L., Davletshin A., Li Z., You J., Tan S. (2020). Application of Polysaccharide Biopolymer in Petroleum Recovery. Polymers.

[51] Agi A., Junin R., Gbonhinbor J., Onyekonwu M. (2018). Natural polymer flow behaviour in porous media for enhanced oil recovery applications: A review. J. Pet. Explor. Prod. Technol..

[52] Irzhak V.I., Uflyand I.E., Dzhardimalieva G.I. (2022). Self-Healing of Polymers and Polymer Composites. Polymers.

[53] Kang C., Guo J., Kiyingi W., Li J., Xue P. (2025). A New Self-Healing Green Polymer Gel with Dynamic Networks for Flow Control in Harsh Reservoirs. Adv. Funct. Mater..

[54] Kim J.R., Netravali A.N. (2018). Self-healing green polymers and composites. Advanced Green Composites.

[55] Kang W., Kang X., Lashari Z.A., Li Z., Zhou B., Yang H., Sarsenbekuly B., Aidarova S. (2021). Progress of polymer gels for conformance control in oilfield. Adv. Colloid Interface Sci..

[56] Zhu D., Bai B., Hou J. (2017). Polymer Gel Systems for Water Management in High-Temperature Petroleum Reservoirs: A Chemical Review. Energy Fuels.

[57] Aldhaheri M., Wei M., Zhang N., Bai B. (2020). Field design guidelines for gel strengths of profile-control gel treatments based on reservoir type. J. Pet. Sci. Eng..

[58] Vargas-Vasquez S.M., Romero-Zerón L.B. (2008). A Review of the Partly Hydrolyzed Polyacrylamide Cr(III) Acetate Polymer Gels. Pet. Sci. Technol..

[59] Vossoughi S. (2000). Profile modification using in situ gelation technology—A review. J. Pet. Sci. Eng..

[60] Pu W., Wen C., Liu R., Jin F., Wang C., Liao Z. (2016). Evaluation of a novel profile control agent for enhancing an oil-recovery application. J. Appl. Polym. Sci..

[61] EC (2006). Registration, Evaluation, Authorization and Restriction of Chemicals (REACH). regulation (EC) no. 1907/2006 of the European Parliament and of the Council. Off. J. Eur. Commun.

[62] Reddy B.R., Eoff L., Dalrymple E.D., Black K., Brown D., Rietjens M. (2003). A Natural Polymer-Based Cross-Linker System for Conformance Gel Systems. Spe J..

[63] China, Ministry of Environmental Protection (2020). Order No. 12 of the Ministry of Environmental Protection of the People’s Republic of China: Measures for the Environmental Management of New Chemical Substances. http://www.gov.cn.

[64] Wang H., Yan Z.-G., Li H., Yang N.-Y., Leung K.M.Y., Wang Y.-Z., Yu R.-Z., Zhang L., Wang W.-H., Jiao C.-Y. (2012). Progress of environmental management and risk assessment of industrial chemicals in China. Environ. Pollut..

[65] Correia M.G., Maschio C., Schiozer D.J. (2017). Development of complex layered and fractured reservoir models for reservoir simulation. J. Braz. Soc. Mech. Sci. Eng..

[66] Amirsardari M., Dabir B., Naderifar A. (2016). Development of a flow based dynamic gridding approach for fluid flow modeling in heterogeneous reservoirs. J. Nat. Gas Sci. Eng..

[67] Li Y., Luo H.W., Li H.T., Liu X.J., Tan Y.S., Chen S.N., Cai J.C. (2020). A brief review of dynamic capillarity effect and its characteristics in low permeability and tight reservoirs. J. Pet. Sci. Eng..

[68] Piepenbrock M.-O.M., Lloyd G.O., Clarke N., Steed J.W. (2010). Metal- and Anion-Binding Supramolecular Gels. Chem. Rev..

[69] Needham R.B., Threlkeld C.B., Gall J.W. Control of Water Mobility Using Polymers and Multivalent Cations. Proceedings of the SPE Improved Oil Recovery Symposium.

[70] Fang J., Zhang X., Li L., Zhang J., Shi X., Hu G. (2023). Research Progress of High-Temperature Resistant Functional Gel Materials and Their Application in Oil and Gas Drilling. Gels.

[71] Afolabi R.O., Oluyemi G.F., Officer S., Ugwu J.O. (2019). Hydrophobically associating polymers for enhanced oil recovery—Part A: A review on the effects of some key reservoir conditions. J. Pet. Sci. Eng..

[72] Chen M., Zhou Z., Yang G., Lü H., Xia X. (2015). Research on feasibility of amphiphilic polymer for chemical flooding in heterogeneous heavy oil reservoir. Pet. Geol. Recovery Effic..

[73] Candau F., Selb J. (1999). Hydrophobically-modified polyacrylamides prepared by micellar polymerization. Adv. Colloid Interface Sci..

[74] Ida S., Nishisako D., Fujiseki A., Kanaoka S. (2021). Thermoresponsive properties of polymer hydrogels induced by copolymerization of hydrophilic and hydrophobic monomers: Comprehensive study of monomer sequence and water affinity. Soft Matter.

[75] Vázquez B., San Roman J., Peniche C., Cohen M.E. (1997). Polymeric Hydrophilic Hydrogels with Flexible Hydrophobic Chains. Control of the Hydration and Interactions with Water Molecules. Macromolecules.

[76] Chen L., Zhu X., Fu M., Zhao H., Li G., Zuo J. (2019). Experimental study of calcium-enhancing terpolymer hydrogel for improved oil recovery in ultrodeep carbonate reservoir. Colloids Surf. A Physicochem. Eng. Asp..

[77] Su X., Hao D., Xu X., Guo X., Li Z., Jiang L. (2020). Hydrophilic/Hydrophobic Heterogeneity Anti-Biofouling Hydrogels with Well-Regulated Rehydration. ACS Appl. Mater. Interfaces.

[78] Biswas S., Singh A., Beziau A., Kowalewski T., Matyjaszewski K., Balazs A.C. (2017). Modeling the formation of layered, amphiphilic gels. Polymer.

[79] Patrickios C.S., Georgiou T.K. (2003). Covalent amphiphilic polymer networks. Curr. Opin. Colloid Interface Sci..

[80] Gumerov R.A., Gau E., Xu W., Melle A., Filippov S.A., Sorokina A.S., Wolter N.A., Pich A., Potemkin I.I. (2020). Amphiphilic PVCL/TBCHA microgels: From synthesis to characterization in a highly selective solvent. J. Colloid Interface Sci..

[81] Sarsenbekuly B., Kang W., Fan H., Yang H., Dai C., Zhao B., Aidarova S.B. (2017). Study of salt tolerance and temperature resistance of a hydrophobically modified polyacrylamide based novel functional polymer for EOR. Colloids Surf. A Physicochem. Eng. Asp..

[82] Yang H.B., Iqbal M.W., Lashari Z.A., Cao C.X., Tang X.C., Kang W.L. (2019). Experimental research on amphiphilic polymer/organic chromium gel for high salinity reservoirs. Colloid Surf. A-Physicochem. Eng. Asp..

[83] Zhang H.W., Yang H.B., Zhou B.B., Li X.X., Zhao H., Wang F., Kang W.L., Sarsenbekuly B., Aidarova S., Gabdullin M. (2020). Effects of cyclodextrin polymer on the gelation of amphiphilic polymer in inclusion complex. J. Mol. Liq..

[84] Singh R., Kamla K., and Mahto V. (2015). Study of the Gelation and Rheological Behavior of Carboxymethyl Cellulose-Polyacrylamide Graft Copolymer Hydrogel. J. Dispers. Sci. Technol..

[85] Díez-Pascual A.M. (2019). Nanoparticle Reinforced Polymers. Polymers.

[86] Awasthi S., Gaur J.K., Bobji M.S., Srivastava C. (2022). Nanoparticle-reinforced polyacrylamide hydrogel composites for clinical applications: A review. J. Mater. Sci..

[87] Agrawal S.K., Sanabria-DeLong N., Tew G.N., Bhatia S.R. (2008). Nanoparticle-Reinforced Associative Network Hydrogels. Langmuir.

[88] Das S., Irin F., Ma L., Bhattacharia S.K., Hedden R.C., Green M.J. (2013). Rheology and Morphology of Pristine Graphene/Polyacrylamide Gels. ACS Appl. Mater. Interfaces.

[89] Yang J., Han C.-R., Duan J.-F., Xu F., Sun R.-C. (2013). Interaction of Silica Nanoparticle/Polymer Nanocomposite Cluster Network Structure: Revisiting the Reinforcement Mechanism. J. Phys. Chem. C.

[90] Meng X., Qiao Y., Do C., Bras W., He C., Ke Y., Russell T.P., Qiu D. (2022). Hysteresis-Free Nanoparticle-Reinforced Hydrogels. Adv. Mater..

[91] Zhou B., Yang H., Li X., Li Z., Bauyrzhan S., Ning C., Shen J., Wang H., Jiang H., Kang W. (2023). Strong thickening performance and mechanism of supramolecular system constructed by β-cyclodextrin polymer included adamantane polymer. J. Mol. Liq..

[92] Rasouli S., Hashemianzadeh S.M. (2023). Thermal behavior of cyclodextrin/adamantane host/guest inclusion complex in an aqueous media. J. Mol. Liq..

[93] Cheng J., Yang H., Gao J., Gu X., Yu X., Su G., Jiang Z., Zhu Y. (2023). Synthesis and molecular dynamics simulation of amphoteric hydrophobically associating polymer. J. Mol. Liq..

[94] Singh R., Mahto V., Vuthaluru H. (2018). Development of a novel fly ash-polyacrylamide nanocomposite gel system for improved recovery of oil from heterogeneous reservoir. J. Pet. Sci. Eng..

[95] Lowe A.B., McCormick C.L. (2002). Synthesis and Solution Properties of Zwitterionic Polymers. Chem. Rev..

[96] Ntente C., Iatridi Z., Theodoropoulou M., Bokias G., Tsakiroglou C. (2023). Anionic amphiphilic copolymers as potential agents for enhanced oil recovery. React. Funct. Polym..

[97] Yang J.-B., Sun J.-S., Bai Y.-R., Lv K.-H., Li J., Li M.-C., Zhu Y.-C. (2023). Preparation and characterization of supramolecular gel suitable for fractured formations. Pet. Sci..

[98] Yang H., Lv Z., Zhang M., Jiang J., Xu B., Shen J., Jiang H., Kang W. (2023). A novel active amphiphilic polymer for enhancing heavy oil recovery: Synthesis, characterization and mechanism. J. Mol. Liq..

[99] Pandit Y.K., Kumar A., Mahto V., Gopalakrishnan Nair U., Matey S., Dhandi M. (2024). Experimental Investigation of a Novel Alumina Nanomaterial Reinforced Particle Gel System for Water Shut-off Jobs in Heterogeneous Reservoirs: Fabrication, Characterization, and Performance Assessment. Ind. Eng. Chem. Res..

[100] Saha R., Nandi R., Saha B. (2011). Sources and toxicity of hexavalent chromium. J. Coord. Chem..

[101] Xing J., Li J., Yang F., Fu Y., Huang J., Bai Y., Bai B. (2022). Cyclic enrichment of chromium based on valence state transformation in metal-free photocatalytic reductive imprinted composite hydrogel. Sci. Total Environ..

[102] Kimbrough D.E., Cohen Y., Winer A.M., Creelman L., Mabuni C. (1999). A Critical Assessment of Chromium in the Environment. Crit. Rev. Environ. Sci. Technol..

[103] Barnhart J. (1997). Chromium chemistry and implications for environmental fate and toxicity. J. Soil Contam..

[104] Dastidar P., Ganguly S., Sarkar K. (2016). Metallogels from Coordination Complexes, Organometallic, and Coordination Polymers. Chem. Asian J..

[105] Lockhart T.P. (1994). Chemical Properties of Chromium/Polyacrylamide Gels. SPE Adv. Technol. Ser..

[106] Yi G., Sayer M. (1996). An acetic acid/water based sol-gel PZT process I: Modification of Zr and Ti alkoxides with acetic acid. J. Sol-Gel Sci. Technol..

[107] Yi G., Sayer M. (1996). An acetic acid/water based sol-gel PZT process II: Formation of a water based solution. J. Sol-Gel Sci. Technol..

[108] Kang C.-H., Guo J.-X., Fei D.-T., Kiyingi W. (2024). Intelligent responsive self-assembled micro-nanocapsules: Used to delay gel gelation time. Pet. Sci..

[109] Mumallah N.A. (1988). Chromium (III) Propionate: A Crosslinking Agent for Water-Soluble Polymers in Hard Oilfield Brines. SPE Reserv. Eng..

[110] Kaddouri A., Mazzocchia C. (2002). Thermoanalytic study of some metal propionates synthesised by sol–gel route: A kinetic and thermodynamic study. J. Anal. Appl. Pyrolysis.

[111] Gel B.A. (1993). Boric Acid Gel. https://www.sigmaaldrich.cn/deepweb/assets/sigmaaldrich/product/documents/312/932/al_techbull_al102.pdf.

[112] Shah S.N., Lord D.L., Rao B.N. Borate-Crosslinked Fluid Rheology Under Various pH, Temperature, and Shear History Conditions. Proceedings of the SPE Production Operations Symposium.

[113] Brannon H.D., Ault M.G. New, Delayed Borate-Crosslinked Fluid Provides Improved Fracture Conductivity in High-Temperature Applications. Proceedings of the SPE Annual Technical Conference and Exhibition.

[114] Harris P.C. (1993). Chemistry and Rheology of Borate-Crosslinked Fluids at Temperatures to 300F. J. Pet. Technol..

[115] de Jong S.J., van Eerdenbrugh B., van Nostrum C.F., Kettenes-van den Bosch J.J., Hennink W.E. (2001). Physically crosslinked dextran hydrogels by stereocomplex formation of lactic acid oligomers: Degradation and protein release behavior. J. Control. Release.

[116] Niu C., Zhang N., Hu C., Zhang C., Zhang H., Xing Y. (2021). Preparation of a novel citric acid-crosslinked Zn-MOF/chitosan composite and application in adsorption of chromium(VI) and methyl orange from aqueous solution. Carbohydr. Polym..

[117] Takahashi R., Sato S., Sodesawa T., Suzuki M., Ichikuni N. (2003). Ni/SiO_2_ prepared by sol–gel process using citric acid. Microporous Mesoporous Mat..

[118] Shibayama M., Shudo Y., Izumi A. (2019). Phenolic Resins—Recent Progress of Structure and Properties Investigations. Macromol. Symp..

[119] Ran Y., Zhang G., Jiang P., Pei H. (2023). Study on Water-Soluble Phenolic Resin Gels for High-Temperature and High-Salinity Oil Reservoir. Gels.

[120] Nichifor M. (2023). Role of Hydrophobic Associations in Self-Healing Hydrogels Based on Amphiphilic Polysaccharides. Polymers.

[121] Gao B., Guo H., Wang J., Zhang Y. (2008). Preparation of hydrophobic association polyacrylamide in a new micellar copolymerization system and its hydrophobically associative property. Macromolecules.

[122] Jiang H., Duan L., Ren X., Gao G. (2019). Hydrophobic association hydrogels with excellent mechanical and self-healing properties. Eur. Polym. J..

[123] Kakuta T., Takashima Y., Harada A. (2013). Highly elastic supramolecular hydrogels using host–guest inclusion complexes with cyclodextrins. Macromolecules.

[124] You Q., Zhang P., Bai S., Huang W., Jia Z., Zhou C., Li D. (2015). Supramolecular linear polymer formed by host–guest interactions of β-cyclodextrin dimers and polyacrylamide end-capped with adamantane. Colloids Surf. A Physicochem. Eng. Asp..

[125] Tungala K., Kumar K., Sonker E., Krishnamoorthi S. (2020). Micellization of amphiphilic host–guest inclusion complexes of polymers based on β–cyclodextrin trimer and adamantane. React. Funct. Polym..

[126] Chen J.H., Rong M.Z., Ruan W.H., Zhang M.Q. (2009). Interfacial enhancement of nano-SiO_2_/polypropylene composites. Compos. Sci. Technol..

[127] Dellatolas I., Bantawa M., Damerau B., Guo M., Divoux T., Del Gado E., Bischofberger I. (2023). Local Mechanism Governs Global Reinforcement of Nanofiller-Hydrogel Composites. ACS Nano.

[128] Bhattacharya S., Samanta S.K. (2016). Soft-Nanocomposites of Nanoparticles and Nanocarbons with Supramolecular and Polymer Gels and Their Applications. Chem. Rev..

[129] Debertrand L., Zhao J., Creton C., Narita T. (2021). Swelling and Mechanical Properties of Polyacrylamide-Derivative Dual-Crosslink Hydrogels Having Metal–Ligand Coordination Bonds as Transient Crosslinks. Gels.

[130] te Nijenhuis K., Mensert A., Zitha P.L.J. (2003). Viscoelastic behaviour of partly hydrolysed polyacrylamide/chromium(III) gels. Rheol. Acta.

[131] Zhang X., Zhang S., Li L., Wu R., Liu D., Wu J., Wu W. (2015). High-temperature-resistant polymer gel system with metal–organic mixed cross-linking agents. J. Appl. Polym. Sci..

[132] Cai W., Huang R. (2001). Study on gelation of partially hydrolyzed polyacrylamide with titanium(IV). Eur. Polym. J..

[133] Kedir A.S., Seland J.G., Skauge A., Skauge T. (2014). Nanoparticles for Enhanced Oil Recovery: Influence of pH on Aluminum-Cross-linked Partially Hydrolyzed Polyacrylamide-Investigation by Rheology and NMR. Energy Fuels.

[134] Karatum O., Bhuiya M.M.H., Carroll M.K., Anderson A.M., Plata D.L. (2018). Life Cycle Assessment of Aerogel Manufacture on Small and Large Scales: Weighing the Use of Advanced Materials in Oil Spill Remediation. J. Ind. Ecol..

[135] Garrido R., Silvestre J.D., Flores-Colen I. (2017). Economic and Energy Life Cycle Assessment of aerogel-based thermal renders. J. Clean. Prod..

[136] Pinto I., Silvestre J.D., de Brito J., Júlio M.F. (2020). Environmental impact of the subcritical production of silica aerogels. J. Clean. Prod..

[137] Turhan Kara I., Kiyak B., Colak Gunes N., Yucel S. (2024). Life cycle assessment of aerogels: A critical review. J. Sol-Gel Sci. Technol..

[138] Enríquez-Martínez V., Niembro-García I.J., Marmolejo-Saucedo J.A. (2021). A Life Cycle Assessment (LCA) of Antibacterial Gel Production. Proceedings of the Computer Science and Engineering in Health Services.

[139] Silva D.A.L., de Oliveira J.A., Lopes Silva D.A., Puglieri F.N., Saavedra Y.M.B. (2021). Life Cycle Assessment (LCA)—Definition of Goals and Scope. Life Cycle Engineering and Management of Products: Theory and Practice.

[140] Leroy-Parmentier N., Valdivia S., Sonnemann G. (2024). Defining the goal and scope for life cycle sustainability assessment. Handbook on Life Cycle Sustainability Assessment.

[141] Toffoletto L., Bulle C., Godin J., Reid C., Deschênes L. (2007). LUCAS—A New LCIA Method Used for a Canadian-Specific Context. Int. J. Life Cycle Assess..

[142] Reddy B.R., Eoff L., Crespo F., Lewis C. Recent Advances in Organically Crosslinked Conformance Polymer Systems. Proceedings of the SPE International Symposium on Oilfield Chemistry.

[143] Hutchins R.D., Dovan H.T., Sandiford B.B. Field Applications of High Temperature Organic Gels for Water Control. Proceedings of the SPE/DOE Improved Oil Recovery Symposium.

[144] Salgaonkar L., Das P. Laboratory Evaluation of Organically Crosslinked Polymer for Water Shutoff in High-Temperature Well Applications. Proceedings of the SPE Kuwait International Petroleum Conference and Exhibition.

[145] Shehbaz S.M., Bera A. (2023). Effects of nanoparticles, polymer and accelerator concentrations, and salinity on gelation behavior of polymer gel systems for water shut-off jobs in oil reservoirs. Pet. Res..

[146] Duceac I.A., Coseri S. (2022). Chitosan Schiff-Base Hydrogels—A Critical Perspective Review. Gels.

[147] Hu S., Ding M., Hu Y., Wang Y., Dong J. (2023). Optimization of the Methods to Develop Stable Polymer Gels for Water Management in Medium- and Ultra-High-Salinity Reservoirs. Gels.

[148] Castro-Muñoz R., Boczkaj G. (2025). Paving the way for green cross-linker substances for the fabrication of polymer membranes—A review. Curr. Opin. Chem. Eng..

[149] Yang J., Li W., Zhu Q., Yang M., Li J., Zhang J., Yang B., Zhao X. (2019). Identification, Formation, and Predicted Toxicity of Halogenated DBPs Derived from Tannic Acid and Its Biodegradation Products. Environ. Sci. Technol..

[150] Gan D., Xing W., Jiang L., Fang J., Zhao C., Ren F., Fang L., Wang K., Lu X. (2019). Plant-inspired adhesive and tough hydrogel based on Ag-Lignin nanoparticles-triggered dynamic redox catechol chemistry. Nat. Commun..

[151] Guo H., Ge J., Li L., Liu M., Wang W. (2024). Development, evaluation and stability mechanism of high-strength gels in high-temperature and high-salinity reservoirs. J. Mol. Liq..

[152] Liu Y., Song S., Liu S., Zhu X., Wang P. (2022). Application of Nanomaterial in Hydrogels Related to Wound Healing. J. Nanomater..

[153] Frazar E.M., Shah R.A., Dziubla T.D., Hilt J.Z. (2020). Multifunctional temperature—Responsive polymers as advanced biomaterials and beyond. J. Appl. Polym. Sci..

[154] Soradech S., Williams A.C., Khutoryanskiy V.V. (2022). Physically Cross-Linked Cryogels of Linear Polyethyleneimine: Influence of Cooling Temperature and Solvent Composition. Macromolecules.

[155] Lim H.-S., Chae S., Yan L., Li G., Feng R., Shin Y., Nie Z., Sivakumar B.M., Zhang X., Liang Y. (2022). Crosslinked Polyethyleneimine Gel Polymer Interface to Improve Cycling Stability of RFBs. Energy Mater. Adv..

[156] Jia H., Chen H. (2018). Using DSC technique to investigate the non-isothermal gelation kinetics of the multi-crosslinked Chromium acetate (Cr3+)-Polyethyleneimine (PEI)-Polymer gel sealant. J. Pet. Sci. Eng..

[157] Duquette D., Dumont M.-J. (2019). Comparative studies of chemical crosslinking reactions and applications of bio-based hydrogels. Polym. Bull..

[158] Nita L.E., Ghilan A., Rusu A.G., Neamtu I., Chiriac A.P. (2020). New Trends in Bio-Based Aerogels. Pharmaceutics.

[159] Pattanayak R., Jena T., Pradhan S., Mohanty S. (2023). Recent advancement of bio-based super absorbent polymer and its biodegradable and recycling behavior: A vision and future. Polym.-Plast. Technol. Mater..

[160] Motornov M., Roiter Y., Tokarev I., Minko S. (2010). Stimuli-responsive nanoparticles, nanogels and capsules for integrated multifunctional intelligent systems. Prog. Polym. Sci..

[161] Takashima Y., Yonekura K., Koyanagi K., Iwaso K., Nakahata M., Yamaguchi H., Harada A. (2017). Multifunctional Stimuli-Responsive Supramolecular Materials with Stretching, Coloring, and Self-Healing Properties Functionalized via Host–Guest Interactions. Macromolecules.

[162] Yang Y., Li R., Gao C., Qin Z., Mi H.-Y., Dong B., Jing X., Liu C., Shen C. (2024). Impact-resistant, high-toughness, self-healable elastomers with physical-chemical dual-crosslinking networks for efficient energy absorption. Appl. Mater. Today.

[163] Li Q.-Q., Xu D., Dong Q.-W., Song X.-J., Chen Y.-B., Cui Y.-L. (2024). Biomedical potentials of alginate via physical, chemical, and biological modifications. Int. J. Biol. Macromol..

[164] An X., Ma C., Gong L., Liu C., Li N., Liu Z., Li X. (2024). Ionic-physical–chemical triple cross-linked all-biomass-based aerogel for thermal insulation applications. J. Colloid Interface Sci..

[165] Becker G., Wurm F.R. (2018). Functional biodegradable polymers via ring-opening polymerization of monomers without protective groups. Chem. Soc. Rev..

[166] Okada M. (2002). Chemical syntheses of biodegradable polymers. Prog. Polym. Sci..

[167] Chen J.-H., Yuan W.-Q., Li Y.-D., Weng Y.-X., Zeng J.-B. (2019). Malleable and Sustainable Poly(ester amide) Networks Synthesized via Melt Condensation Polymerization. ACS Sustain. Chem. Eng..

[168] Qu T., West K.N., Rupar P.A. (2023). Rapid synthesis of functional poly (ester amide) s through thiol–ene chemistry. RSC Adv..

[169] Wei Z., He J., Liang T., Oh H., Athas J., Tong Z., Wang C., Nie Z. (2013). Autonomous self-healing of poly (acrylic acid) hydrogels induced by the migration of ferric ions. Polym. Chem..

[170] Liu G., Zou F., He W., Li J., Xie Y., Ma M., Zheng Y. (2023). The controlled degradation of bacterial cellulose in simulated physiological environment by immobilization and release of cellulase. Carbohydr. Polym..

[171] Ginjupalli K., Shavi G.V., Averineni R.K., Bhat M., Udupa N., Nagaraja Upadhya P. (2017). Poly(α-hydroxy acid) based polymers: A review on material and degradation aspects. Polym. Degrad. Stab..

[172] Yao F., Bai Y., Chen W., An X., Yao K., Sun P., Lin H. (2004). Synthesis and characterization of functional l-lactic acid/citric acid oligomer. Eur. Polym. J..

[173] Yao F., Chen W., Liu C., De Yao K. (2003). A novel amphoteric, pH-sensitive, biodegradable poly [chitosan-g-(l-lactic-co-citric) acid] hydrogel. J. Appl. Polym. Sci..

[174] Alvarez-Lorenzo C., Concheiro A. (2002). Reversible adsorption by a pH- and temperature-sensitive acrylic hydrogel. J. Control. Release.

[175] Osada Y., Gong J. (1993). Stimuli-responsive polymer gels and their application to chemomechanical systems. Prog. Polym. Sci..

[176] Goponenko A.V., Dzenis Y.A. (2016). Role of mechanical factors in applications of stimuli-responsive polymer gels—Status and prospects. Polymer.

[177] Nakajima T., Sato H., Zhao Y., Kawahara S., Kurokawa T., Sugahara K., Gong J.P. (2012). A Universal Molecular Stent Method to Toughen any Hydrogels Based on Double Network Concept. Adv. Funct. Mater..

[178] Haque M.A., Kurokawa T., Gong J.P. (2012). Super tough double network hydrogels and their application as biomaterials. Polymer.

[179] Xin H. (2022). Double-Network Tough Hydrogels: A Brief Review on Achievements and Challenges. Gels.

[180] Zhang M., Choi W., Kim M., Choi J., Zang X., Ren Y., Chen H., Tsukruk V., Peng J., Liu Y. (2024). Recent advances in environmentally friendly dual-crosslinking polymer networks. Angew. Chem..

[181] Zhang H., Shi L.W.E., Zhou J. (2023). Recent developments of polysaccharide-based double-network hydrogels. J. Polym. Sci..

[182] Yang H., van Ruymbeke E., Fustin C.-A. (2022). Influence of Network Topology on the Viscoelastic Properties of Double Dynamics Hydrogels. Macromolecules.

[183] Rodin M., Li J., Kuckling D. (2021). Dually cross-linked single networks: Structures and applications. Chem. Soc. Rev..

[184] Zhang D., Chen Q., Chen H., Tang Y., Zheng J. (2024). Spontaneous Macrophase Separation Strategy for Bridging Hydrogels from Bilayer to Double-Network Structure. Acc. Mater. Res..

[185] Tominaga T., Tirumala V.R., Lee S., Lin E.K., Gong J.P., Wu W.-l. (2008). Thermodynamic Interactions in Double-Network Hydrogels. J. Phys. Chem. B.

[186] Yang J., Chen Y., Zhao L., Zhang J., Luo H. (2023). Constructions and Properties of Physically Cross-Linked Hydrogels Based on Natural Polymers. Polym. Rev..

[187] Šomvársky J., te Nijenhuis K., Ilavský M. (2000). Polyfunctional Cross-Linking of Existing Polymer Chains. Macromolecules.

[188] Andrianov K.A., Emel’yanov V.N. (1976). Some Aspects of the Theory of Gel Formation in Reactions of Polyfunctional Compounds. Russ. Chem. Rev..

[189] Wang X., Li C., Shi Z., Zhi M., Hong Z. (2018). The investigation of an organic acid assisted sol–gel method for preparing monolithic zirconia aerogels. RSC Adv..

[190] Karoyo A.H., Wilson L.D. (2021). A Review on the Design and Hydration Properties of Natural Polymer-Based Hydrogels. Materials.

[191] Kumar G., Bristow J.F., Smith P.J., Payne G.F. (2000). Enzymatic gelation of the natural polymer chitosan. Polymer.

[192] Brotherton E. (2022). Synthesis and Applications of New Hydrophilic Aldehyde-Functional Methacrylic Polymers. Doctoral Dissertation.

[193] Dong S., He L., Li L., Wu Y., Wang X. (2023). Investigation of Polyvinyl Alcohol–Phenolic Aldehyde–Polyacrylamide Gel for the Application in Saline Oil Reservoirs for Profile Modification. Energy Fuels.

[194] Brotherton E.E., Jesson C.P., Warren N.J., Smallridge M.J., Armes S.P. (2021). New aldehyde-functional methacrylic water-soluble polymers. Angew. Chem..

[195] Karlsson S., Backlund S., Eriksson F., Hedström G. (1998). Enzymatic esterifications and transesterifications in AOT-based gels with different composition. Colloids Surf. B Biointerfaces.

[196] Matsuda F., Miyamoto S., Iizawa T. (1999). Novel Synthesis of Gel Capsule by Esterification of Poly(acrylic acid) Gel. Polym. J..

[197] Sun S., Sun P., Liu D. (2005). The study of esterifying reaction between epoxy resins and carboxyl acrylic polymers in the presence of tertiary amine. Eur. Polym. J..

[198] Li X., Wang M., Liu Z., Yang S., Xu N., Zhao W., Luo G., Liu S. (2024). Alleviation of the plastic deformation of gel ink under strong stress through an esterification of xanthan gum reinforcing its double helix structure. Chin. J. Chem. Eng..

[199] Rivas M.V., Muñetón M.J.A., Bordoni A.V., Lombardo M.V., Spagnuolo C.C., Wolosiuk A. (2023). Revisiting carboxylic group functionalization of silica sol–gel materials. J. Mater. Chem. B.

[200] Wurm F., Rietzler B., Pham T., Bechtold T. (2020). Multivalent Ions as Reactive Crosslinkers for Biopolymers—A Review. Molecules.

[201] Perić-Hassler L., Hünenberger P.H. (2010). Interaction of alginate single-chain polyguluronate segments with mono-and divalent metal cations: A comparative molecular dynamics study. Mol. Simul..

[202] Tavakoli V. (2019). Carbonate Reservoir Heterogeneity: Overcoming the Challenges.

[203] Duan R., Xu Z., Dong Y., Liu W. (2021). Characterization and classification of pore structures in deeply buried carbonate rocks based on mono- and multifractal methods. J. Pet. Sci. Eng..

[204] Cicha-Szot R., Labus K., Leśniak G. (2025). Pore-Scale Evolution of Carbonate and Sandstone Reservoirs Under CO2–Brine Interaction: Implications for Sustainable Carbon Storage. Sustainability.

[205] Gao Y., Li T., Zhang Z., Yu J., Zhang Y., Li X., Zhao H. (2023). Research on fluid mobility in tight-sandstone with a NMR fractal theory pore classification method. Front. Earth Sci..

[206] Li M., Qu Z., Wang M., Ran W. (2023). The Influence of Micro-Heterogeneity on Water Injection Development in Low-Permeability Sandstone Oil Reservoirs. Minerals.

[207] Mohammadmoradi P., Bashtani F., Goudarzi B., Taheri S., Kantzas A. Pore Network and Morphological Characterization of Pore-Level Structures. Proceedings of the SPE Canada Heavy Oil Technical Conference.

[208] Wang C., Liu X., Wang E., Wang M., Liu C. (2023). Dependence of connectivity dominance on fracture permeability and influence of topological centrality on the flow capacity of fractured porous media. J. Hydrol..

[209] Sarkar P., Singh K.H., Singh T.N., Ghosh R., Sarkar P., Singh K.H., Singh T.N., Ghosh R. (2025). Pore Topology. Laboratory Characterization of Shale: Measurement and Simulation.

[210] Stoop N., Waisbord N., Kantsler V., Heinonen V., Guasto J.S., Dunkel J. (2019). Disorder-induced topological transition in porous media flow networks. J. Non-Newton. Fluid Mech..

[211] Wang M., Cui Z., Xue Y. (2021). Determination of Interfacial Tension of Nanomaterials and the Effect of Particle Size on Interfacial Tension. Langmuir.

[212] Yu L., Sang Q., Dong M., Yuan Y. (2017). Effects of Interfacial Tension and Droplet Size on the Plugging Performance of Oil-in-Water Emulsions in Porous Media. Ind. Eng. Chem. Res..

[213] von Nessen K., Karg M., Hellweg T. (2013). Thermoresponsive poly-(N-isopropylmethacrylamide) microgels: Tailoring particle size by interfacial tension control. Polymer.

[214] Lepercq-Bost É., Giorgi M.-L., Isambert A., Arnaud C. (2008). Use of the capillary number for the prediction of droplet size in membrane emulsification. J. Membr. Sci..

[215] Style R.W., Hyland C., Boltyanskiy R., Wettlaufer J.S., Dufresne E.R. (2013). Surface tension and contact with soft elastic solids. Nat. Commun..

[216] Mohammadmoradi P., Kantzas A. Pore Scale Investigation of Wettability Effect on Waterflood Performance. Proceedings of the SPE Annual Technical Conference and Exhibition.

[217] Lewis M.G., Sharma M.M., Dunlap H.F. Wettability and Stress Effects on Saturation and Cementation Exponents. Proceedings of the SPWLA 29th Annual Logging Symposium.

[218] Khurshid I., Al-Attar H., Alraeesi A. (2018). Modeling cementation in porous media during waterflooding: Asphaltene deposition, formation dissolution and their cementation. J. Pet. Sci. Eng..

[219] Barclay S.A., Worden R.H. (2000). Effects of Reservoir Wettability on Quartz Cementation in Oil Fields. Quartz Cementation in Sandstones.

[220] Changfu X., Hongxian L., Genbao Q., Jianhua Q. (2011). Microcosmic mechanisms of water-oil displacement in conglomerate reservoirs in Karamay Oilfield, NW China. Pet. Explor. Dev..

[221] Lin Z., Li J., Wang M., Zhang P., Lu S., Zhi Q., Wang J., Huang H. (2022). Organic fluid migration in low permeability reservoirs restricted by pore structure parameters. J. Pet. Sci. Eng..

[222] Vafaie A., Kivi I.R., Moallemi S.A., Habibnia B. (2021). Permeability prediction in tight gas reservoirs based on pore structure characteristics: A case study from South Western Iran. Unconv. Resour..

[223] Mustafa A., Mahmoud M.A., Abdulraheem A. A Review of Pore Structure Characterization of Unconventional Tight Reservoirs. Proceedings of the Abu Dhabi International Petroleum Exhibition & Conference.

[224] Fan C., Cao J., Luo J., Li S., Wu S., Dai L., Hou J., Mao Q. (2021). Heterogeneity and influencing factors of marine gravity flow tight sandstone under abnormally high pressure: A case study from the Miocene Huangliu Formation reservoirs in LD10 area, Yinggehai Basin, South China Sea. Pet. Explor. Dev..

[225] Xiao Z., Ding W., Hao S., Taleghani A.D., Wang X., Zhou X., Sun Y., Liu J., Gu Y. (2019). Quantitative analysis of tight sandstone reservoir heterogeneity based on rescaled range analysis and empirical mode decomposition: A case study of the Chang 7 reservoir in the Dingbian oilfield. J. Pet. Sci. Eng..

[226] Ju Y., Sun Y., Tan J., Bu H., Han K., Li X., Fang L. (2018). The composition, pore structure characterization and deformation mechanism of coal-bearing shales from tectonically altered coalfields in eastern China. Fuel.

[227] Rachinsky M., Kerimov V.Y. (2015). Fluid Dynamics of Oil and Gas Reservoirs.

[228] Mullins O.C., Zuo J.Y., Wang K., Hammond P.S., De Santo I., Dumont H., Mishra V.K., Chen L., Pomerantz A.E., Dong C. (2014). The Dynamics of Reservoir Fluids and their Substantial Systematic Variations. Petrophysics—SPWLA J..

[229] Gussow W.C. (1968). Migration of Reservoir Fluids. J. Pet. Technol..

[230] Liang M., Wang Z., Gao L., Li C., Li H. (2017). Evolution of pore structure in gas shale related to structural deformation. Fuel.

[231] Loucks R.G., Reed R.M., Ruppel S.C., Jarvie D.M. (2009). Morphology, Genesis, and Distribution of Nanometer-Scale Pores in Siliceous Mudstones of the Mississippian Barnett Shale. J. Sediment. Res..

[232] Clarkson C.R., Solano N., Bustin R.M., Bustin A.M.M., Chalmers G.R.L., He L., Melnichenko Y.B., Radliński A.P., Blach T.P. (2013). Pore structure characterization of North American shale gas reservoirs using USANS/SANS, gas adsorption, and mercury intrusion. Fuel.

[233] Clarkson C.R., Haghshenas B., Ghanizadeh A., Qanbari F., Williams-Kovacs J.D., Riazi N., Debuhr C., Deglint H.J. (2016). Nanopores to megafractures: Current challenges and methods for shale gas reservoir and hydraulic fracture characterization. J. Nat. Gas Sci. Eng..

[234] Cappa F., Guglielmi Y., Fénart P., Merrien-Soukatchoff V., Thoraval A. (2005). Hydromechanical interactions in a fractured carbonate reservoir inferred from hydraulic and mechanical measurements. Int. J. Rock Mech. Min. Sci..

[235] Wei M., Dai F., Ji Y., Wu W. (2021). Effect of fluid pressure gradient on the factor of safety in rock stability analysis. Eng. Geol..

[236] Cornell D., Katz D.L. (1953). Pressure Gradients in Natural Gas Reservoirs. J. Pet. Technol..

[237] Montel F., Bickert J., Hy-Billiot J., Royer M. Pressure and Compositional Gradients in Reservoirs. Proceedings of the Nigeria Annual International Conference and Exhibition.

[238] Sarma H.K., Bentsen R.G. (1989). A study of the impact of instability on relative permeability and capillary pressure. J. Pet. Sci. Eng..

[239] Abbasi S., Khamehchi E. (2021). Investigation of permeability decline due to coupled precipitation/dissolution mechanism in carbonate rocks during low salinity co-water injection. Energy Rep..

[240] Amour F., Bonto M., Hajiabadi M.R., Nick H.M. Sensitivity Study of Chemical Effects on the Compaction Behavior of Reservoir Chalk (Dan Field, Danish North Sea). Proceedings of the 55th U.S. Rock Mechanics/Geomechanics Symposium.

[241] Zhou L., Wu J., Ji J.-H., Gao J., Liu Y.-F., Wang B., Yang S.-Z., Gu J.-D., Mu B.-Z. (2023). Characteristics of microbiota, core sulfate-reducing taxa and corrosion rates in production water from five petroleum reservoirs in China. Sci. Total Environ..

[242] Mangane P.O., Gouze P., Luquot L. (2013). Permeability impairment of a limestone reservoir triggered by heterogeneous dissolution and particles migration during CO_2_-rich injection. Geophys. Res. Lett..

[243] Zhang S., Fang Z. (2020). Permeability damage micro-mechanisms and stimulation of low-permeability sandstone reservoirs: A case study from Jiyang Depression, Bohai Bay Basin, China. Pet. Explor. Dev..

[244] O’neill T.J. (2003). Life Cycle Assessment and Environmental Impact of Polymeric Products.

[245] Ghosh P., Wilton R.R., Bowers A., O’Brien T., Cao Y., Wilson C., Metidji M.O., Dupuis G., Ravikiran R. Re-Injection of Produced Polymer in EOR Projects to Improve Economics and Reduce Carbon Footprint. Proceedings of the SPE Improved Oil Recovery Conference.

[246] Quintero H.I., Solorzano P.L., Barbosa C., Corredor L.M., Martinez A., Feriz E.F., HernÁNdez R., Vega S.M., Carrasca L K.J., Zapata J.F. Carbon Footprint and Energy Intensity Assessment for Enhanced Oil Recovery (EOR) Based on Polymer Injection: A Colombian Case Study. Proceedings of the SPE Improved Oil Recovery Conference.

[247] Sajid M., Płotka-Wasylka J. (2022). Green analytical chemistry metrics: A review. Talanta.

[248] Tufvesson L.M., Tufvesson P., Woodley J.M., Börjesson P. (2013). Life cycle assessment in green chemistry: Overview of key parameters and methodological concerns. Int. J. Life Cycle Assess..

[249] Sheldon R.A. (2018). Metrics of Green Chemistry and Sustainability: Past, Present, and Future. ACS Sustain. Chem. Eng..

[250] Judson R., Richard A., Dix D.J., Houck K., Martin M., Kavlock R., Dellarco V., Henry T., Holderman T., Sayre P. (2009). The toxicity data landscape for environmental chemicals. Environ. Health Perspect..

[251] Fantke P., Aurisano N., Provoost J., Karamertzanis P.G., Hauschild M. (2020). Toward effective use of REACH data for science and policy. Environ. Int..

[252] Hoffman D.J., Rattner B.A., Burton G.A., Cairns J. (2002). Handbook of Ecotoxicology.

[253] Kim S., Chen J., Cheng T., Gindulyte A., He J., He S., Li Q., Shoemaker B.A., Thiessen P.A., Yu B. (2023). PubChem 2023 update. Nucleic Acids Res..

[254] Ruiz P., Faroon O., Mumtaz M., Wohlers D. (2012). Toxicological Profile for Acrylamide.

[255] Tchounwou P.B., Yedjou C., Patlolla A., Sutton D. (2012). Environmental toxicology. Mol. Clin. Environ. Toxicol. Exp. Suppl..

[256] Ali H., Khan E., Ilahi I. (2019). Environmental Chemistry and Ecotoxicology of Hazardous Heavy Metals: Environmental Persistence, Toxicity, and Bioaccumulation. J. Chem..

[257] Lau M.H.Y., Leung K.M.Y., Wong S.W.Y., Wang H., Yan Z.-G. (2012). Environmental policy, legislation and management of persistent organic pollutants (POPs) in China. Environ. Pollut..

[258] Abelkop A.D., Graham J.D., Royer T.V. (2018). Persistent, Bioaccumulative, and Toxic (PBT) Chemicals: Technical Aspects, Policies, and Practices.

[259] Matthies M., Solomon K., Vighi M., Gilman A., Tarazona J.V. (2016). The origin and evolution of assessment criteria for persistent, bioaccumulative and toxic (PBT) chemicals and persistent organic pollutants (POPs). Environ. Sci. Process. Impacts.

[260] Rücker T. (2024). Safe Chemicals/REACH. Drug Discovery and Evaluation: Safety and Pharmacokinetic Assays.

[261] Prevention P. (2014). TSCA Work Plan Chemical Risk Assessment. https://www.epa.gov/sites/default/files/2015-09/documents/hhcb_wp_ra_final_08_27_14.pdf.

[262] McPartland J., Shaffer R.M., Fox M.A., Nachman K.E., Burke T.A., Denison R.A. (2022). Charting a Path Forward: Assessing the Science of Chemical Risk Evaluations under the Toxic Substances Control Act in the Context of Recent National Academies Recommendations. Environ. Health Perspect..

[263] Elrod A.A. (2022). The EPA and its regulations. The Palgrave Handbook of Global Sustainability.

[264] Braun O., Coquery C., Kieffer J., Blondel F., Favero C., Besset C., Mesnager J., Voelker F., Delorme C., Matioszek D. (2022). Spotlight on the Life Cycle of Acrylamide-Based Polymers Supporting Reductions in Environmental Footprint: Review and Recent Advances. Molecules.

